# Single-cell guided prenatal derivation of primary fetal epithelial organoids from human amniotic and tracheal fluids

**DOI:** 10.1038/s41591-024-02807-z

**Published:** 2024-03-04

**Authors:** Mattia Francesco Maria Gerli, Giuseppe Calà, Max Arran Beesley, Beatrice Sina, Lucinda Tullie, Kylin Yunyan Sun, Francesco Panariello, Federica Michielin, Joseph R. Davidson, Francesca Maria Russo, Brendan C. Jones, Dani Do Hyang Lee, Savvas Savvidis, Theodoros Xenakis, Ian C. Simcock, Anna A. Straatman-Iwanowska, Robert A. Hirst, Anna L. David, Christopher O’Callaghan, Alessandro Olivo, Simon Eaton, Stavros P. Loukogeorgakis, Davide Cacchiarelli, Jan Deprest, Vivian S. W. Li, Giovanni Giuseppe Giobbe, Paolo De Coppi

**Affiliations:** 1 Department of Surgical Biotechnology, Division of Surgery and Interventional Science, University College London, London, UK; 2 Great Ormond Street Institute of Child Health, University College London, London, UK; 3 Politecnico di Milano, Milan, Italy; 4 Stem Cell and Cancer Biology Laboratory, The Francis Crick Institute, London, UK; 5 Armenise/Harvard Laboratory of Integrative Genomics, Telethon Institute of Genetics and Medicine, Pozzuoli, Italy; 6 Elizabeth Garrett Anderson Institute for Women’s Health, University College London, London, UK; 7 Department of Development and Regeneration, Woman and Child and UZ Leuven Clinical Department of Obstetrics and Gynaecology, KU Leuven, Leuven, Belgium; 8 Department of Medical Physics and Biomedical Engineering, University College London, London, UK; 9 Department of Radiology, Great Ormond Street Hospital, London, UK; 10 Department of Respiratory Sciences, University of Leicester, Leicester, UK; 11 Specialist Neonatal and Paediatric Surgery, Great Ormond Street Hospital for Children NHS Foundation Trust, London, UK; 12 Department of Translational Medicine, University of Naples Federico II, Naples, Italy; 13 Genomics and Experimental Medicine Program, Scuola Superiore Meridionale, Naples, Italy; 14 Medical and Surgical Department of the Fetus, Newborn and Infant, Ospedale Pediatrico Bambino Gesù, IRCCS, Rome, Italy; 15 NIHR Great Ormond Street Hospital Biomedical Research Centre, London, UK

**Keywords:** Stem cells, Regenerative medicine, Stem-cell research, Experimental models of disease

## Abstract

Isolation of tissue-specific fetal stem cells and derivation of primary organoids is limited to samples obtained from termination of pregnancies, hampering prenatal investigation of fetal development and congenital diseases. Therefore, new patient-specific in vitro models are needed. To this aim, isolation and expansion of fetal stem cells during pregnancy, without the need for tissue samples or reprogramming, would be advantageous. Amniotic fluid (AF) is a source of cells from multiple developing organs. Using single-cell analysis, we characterized the cellular identities present in human AF. We identified and isolated viable epithelial stem/progenitor cells of fetal gastrointestinal, renal and pulmonary origin. Upon culture, these cells formed clonal epithelial organoids, manifesting small intestine, kidney tubule and lung identity. AF organoids exhibit transcriptomic, protein expression and functional features of their tissue of origin. With relevance for prenatal disease modeling, we derived lung organoids from AF and tracheal fluid cells of congenital diaphragmatic hernia fetuses, recapitulating some features of the disease. AF organoids are derived in a timeline compatible with prenatal intervention, potentially allowing investigation of therapeutic tools and regenerative medicine strategies personalized to the fetus at clinically relevant developmental stages.

## Abstract (web summary)

The generation of primary organoids, from fetal fluid-derived epithelial stem or progenitor cells, offers the possibility of modeling different developing tissues during gestation, even beyond the limits of pregnancy termination.

## Main

Although prenatal diagnosis of congenital anomalies adopts sophisticated genetic and imaging analyses^1,2^, prediction of severity remains challenging, limiting patient-specific parental counseling. Patient stratification for prenatal therapy has shown level 1 evidence for improved outcomes in congenital diaphragmatic hernia (CDH)^3,4^, twin-to-twin transfusion syndrome (TTTS)^5^ and myelomeningocele (MMC)^6^. For other conditions, such as lower urinary tract obstruction (LUTO)^7^, where vesico-amniotic shunting is technically possible, appropriate patient selection remains a hurdle. The lack of autologous models of developing human tissues is a bottleneck to these advancements.

Organoids are three-dimensional (3D) models recapitulating some biological and pathophysiological features of patient’s tissues in vitro. Autologous organoids can be derived from human embryonic stem cells^8^ or through induced pluripotent stem (iPS) cells. iPS cell-derived organoids have been generated from reprogrammed AF-derived fetal cells^9,10^. These organoids resemble fetal-like tissues, but the extensive manipulation and lengthy differentiation protocols reduce patient fidelity and hinder applicability for prenatal disease modeling and targeted therapy.

In contrast, primary organoids, requiring minimal in vitro manipulation, have been derived from human discarded postnatal biological samples (for example, urine, menstrual flow, PAP smear and bronchoalveolar lavage^11–14^). In prenatal medicine, primary organoids were generated from fetal tissues collected postmortem through biobanks^15^; however, accessing fetal tissues is associated with ethico-legal restrictions hampering their research^16–20^. Current methods for primary fetal organoid derivation are destructive, restricting the use for prenatal modeling, diagnostics and regenerative medicine^21,22^. Furthermore, fetal material can be procured mostly up to 20–22 weeks post-conception, making investigation of later developmental stages unfeasible^23^. Here we present derivation of primary human fetal epithelial organoids, of multiple tissue identities, obtained from fetal fluids collected at prenatal diagnostic and therapeutic interventions during the second and third trimesters. This allows generation of organoids alongside continuation of pregnancy.

Around 30,000 amniocenteses are performed annually in the UK^24^ for confirmatory diagnosis and advanced diagnostics^2^. Moreover, amniodrainage is routine treatment for polyhydramnios^25,26^ and TTTS^27^. Finally, prenatal MMC repair and fetoscopic endoluminal tracheal occlusion (FETO) for CDH provide additional access to fetal fluids during pregnancy^3,4,6^. The AF surrounds, supports and protects the fetus during development. Its origin and recirculation follow complex dynamics, progressing together with fetal and extra-embryonic tissue development. AF is highly heterogeneous in origin and composition, containing secretions and cells from various fetal tissues such as the gastrointestinal tract, kidney and lung^28,29^; however, a detailed map of the AF epithelial populations and their potential is lacking. AF harbors stem cells from mesenchymal and hematopoietic niches^30–32^ but most AF cells are epithelial and have only been partially characterized. Using single-cell sequencing, we investigated human AF epithelial cells (AFEpCs), highlighting that these originate from multiple developing tissues. We then explored whether this cell population contains lineage-committed progenitors capable of forming tissue-specific primary fetal organoids. Additionally, we expanded our findings to tracheal fluid (TF) epithelial cells obtained from CDH cases during FETO^33^.

This work demonstrates that diverse epithelial stem cell populations shed into the AF and TF can form epithelial organoids resembling their tissue of origin. Autologous derivation of primary fetal organoids during continuing pregnancies could enable the development of advanced prenatal models and improve counseling and designing personalized therapies. Finally, AF organoids (AFOs) offer the possibility of researching later gestational stages currently inaccessible, being beyond the limits of termination of pregnancy.

## Results

### Single-cell mapping the human AF to investigate presence of tissue-specific fetal epithelial progenitors

**Fig. 1:**
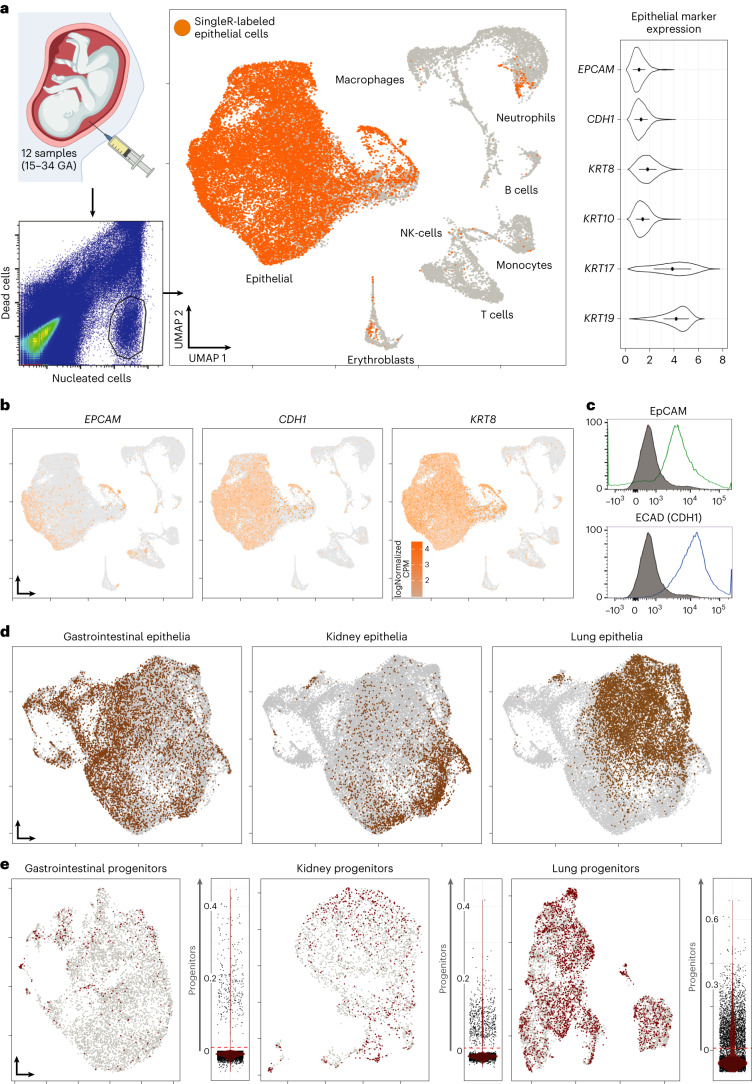
Single-cell analysis of the AF content. **a**, Top left: graphical representation of AF sampling. Bottom left: the FACS plot shows the sorting strategy utilized to collect the living cell fraction, negative for propidium iodide (PI) and positive for Hoechst. Middle: the UMAP shows the content of the AF of multiple patients obtained across the second and third trimesters of pregnancy (*n* = 12 biologically independent AF samples spanning 15–34 GA; 33,934 cells post-filtering examined over 11 sequencing lanes). Highlighted in orange is the epithelial cluster, as identified by the SingleR cell-labeling package using the human primary cell atlas dataset as reference. Right: the violin plots show the level of expression of the pan-epithelial specific genes *EPCAM*, *CDH1(ECAD)*, *KRT8*, *KRT10*, *KRT17* and *KRT19* (mean ± s.d., data presented as normalized counts per million (CPM)). **b**, The UMAPs show the expression of a selection of epithelial markers, within the epithelial cluster identified in **a**. **c**, Representative flow cytometry analysis of EpCAM (*n* = 58,964 cells) and ECAD (CDH1, *n* = 38,389 cells) expression in live-sorted cells from the AF; gray represents unstained control (*n* = 34,045 cells). **d**, Re-calculated UMAP of the epithelial cluster identified in **a**, highlighting cells attributed to the three tissues through scGSEA. **e**, Scoring of the cells identified in **d**, for appropriate progenitor-associated genes. Cells with a positive score are highlighted on a re-calculated UMAP of that tissue’s cells. Scores also plotted as violin plots, identifying distinct populations of progenitor cells, threshold for positive scoring shown in red. Source data

**Extended Data Fig. 1:**
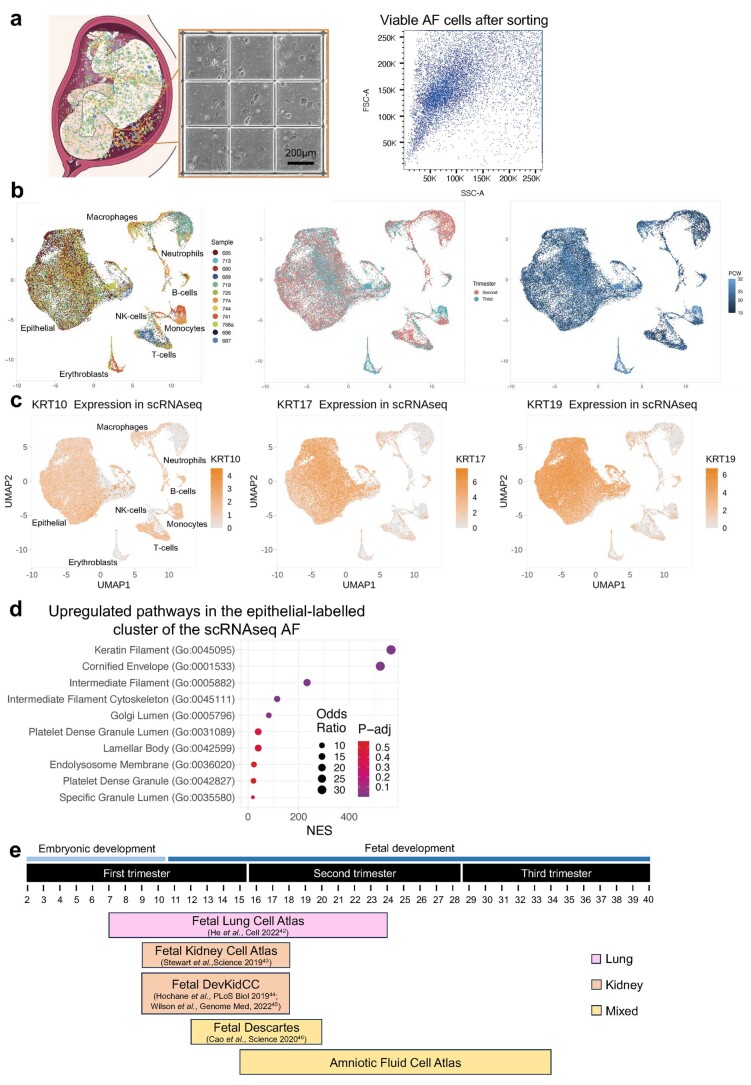
AF single-cell RNA Sequencing. **(a)** Schematic and phase-contrast image of fresh AF sample at collection (left). FACS plot showing heterogeneity of AF cells by forward and side scatter after sorting (right). **(b)** scRNA-seq UMAP analysis of n=12 AF samples; middle UMAP shows cell distribution across second and third trimester; right panel shows cells labeled by post-conception weeks (PCW). **(c)** UMAPs depicting expression of epithelial keratins within the AF epithelial cell cluster. **(d)** Upregulated GO pathways in the epithelial-labeled cluster of the scRNA-seq AF from DEGs calculated when compared to all other clusters (one-sided Fisher test, adjusted using Benjamini-Hochberg for multiple hypotheses). **(e)** Schematic showing the comparison between the gestational age weeks covered by reference fetal scRNA-seq atlases, highlighting the lack of late-stage development data. Source data

We collected AF from 12 pregnancies (Supplementary Tables 1 and 2) and isolated, using fluorescence-activated cell sorting (FACS), the viable nucleated cells with an intact cell membrane (Fig. 1a and Extended Data Fig. 1a). We then performed 3′ single-cell RNA sequencing (scRNA-seq) and generated an unsupervised Uniform Manifold Approximation and Projection (UMAP) using Seurat v.4 (Fig. 1a and Extended Data Fig. 1b). The SingleR package was applied to automatically annotate the epithelial cluster based on primary human cell atlas data^34^, then confirmed by expression of pan-epithelial marker genes (Fig. 1a,b and Extended Data Fig. 1c,d). Protein validation conducted using flow cytometry, confirmed broad presence of epithelial cell adhesion molecule (EpCAM) and ECAD in the majority of viable AF cells (Fig. 1c). We then probed the AFEpC cluster for the presence of specific gastrointestinal, kidney and lung signatures using single-cell gene set enrichment analysis (scGSEA; Fig. 1d). Finally, we scored these cells for canonical tissue-specific progenitor markers: *LGR5*, *OLFM4*, *LRIG1*, *CDX2*, *CD44*, *LYZ*, *SMOC2* and *PROCR* (gastrointestinal); *PAX2*, *PAX8*, *LHX1*, *JAG1*, *SIX2*, *RET*, *HNF4A*, *GATA3*, *POU3F3* and *WT1* (kidney); and *NKX2-1*, *SOX9*, *ETV4*, *ETV5*, *GATA6* and *ID2* (lung). This indicated the presence of gastrointestinal, renal and pulmonary epithelial progenitor cells in AF (Fig. 1e).

### Generation of primary fetal epithelial human AFOs

**Fig. 2:**
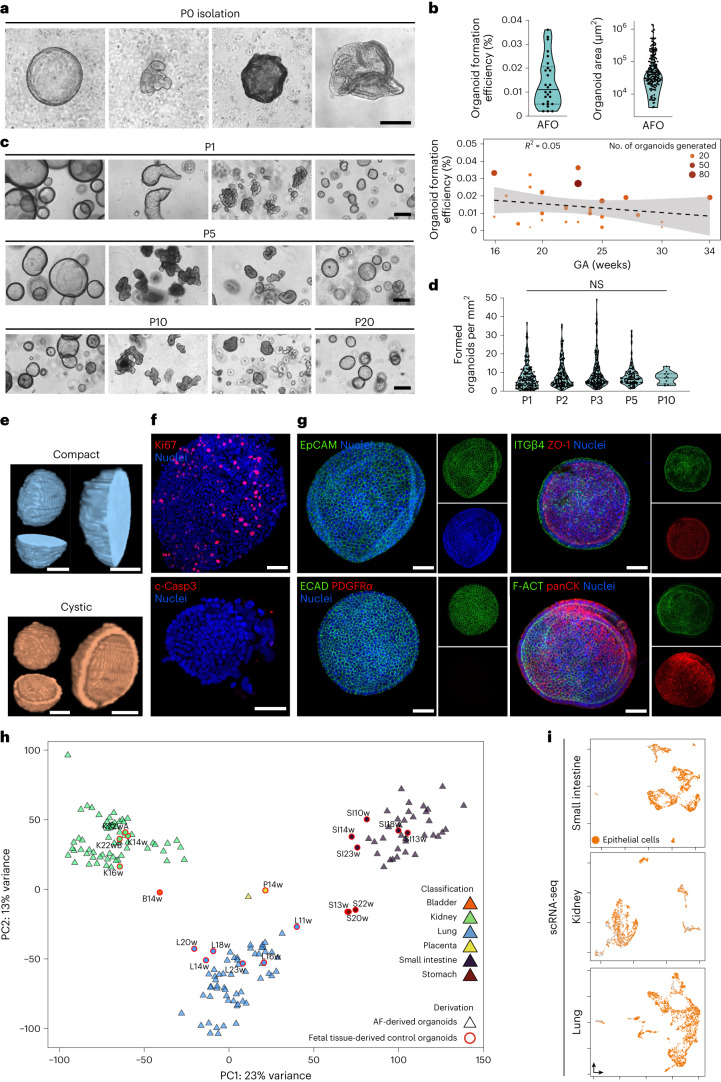
Generation of primary fetal epithelial AFOs. **a**, Phase-contrast images showing organoid formation from 3D cultured viable AF cells, with different organoid morphologies observed at day 14 (scale bar, 200 μm). **b**, Top: formation efficiency (organoids per live cells) and size (organoid area) of AFOs at isolation (passage (P) 0) (*n* = 26 independent AF samples for efficiency plot and *n* = 197 organoids for area plot; median and quartiles for both plots). Bottom: linear regression plot representing organoid formation efficiency (organoids per live cells) at various GAs. Color and size represent the total organoid number generated per sample; dashed line represents linear regression, *R*^2^ = 0.05 and s.e.m. is shown in gray. **c**, Phase-contrast images showing multiple clonal AFO morphologies in expansion, at P1, P5, P10 and up to P20 (scale bars, 200 μm). **d**, Formed organoids per mm^2^ at 7–15 d of culture quantified over ten passages (*n* ≥ 11 organoids from *n* = 19 independent AF samples; median and quartiles; NS, non-significant; one-way analysis of variance (ANOVA) with multiple comparison). **e**, X-ray PC-CT of two organoid phenotypes observed (compact and cystic). Scale bars, 25 μm. **f**, Immunofluorescent staining showing expression of the proliferative marker Ki67 and lack of cleaved caspase 3 apoptotic cells in AFOs at P3; nuclei counterstained with Hoechst (scale bars, 50 μm). **g**, Immunofluorescent staining showing AFO at P3 expressing the epithelial markers EpCAM, ECAD and pan-cytokeratin, while lacking expression of the mesenchymal marker PDGFRɑ. Immunofluorescent staining also shows AFO polarization, highlighted by the presence of the epithelial tight junction ZO-1 on the luminal surface and basolateral ITGβ4. Phalloidin counterstain highlights actin filaments (F-ACT) (scale bars, 50 μm). **h**, Unsupervised PCA plot showing AFOs (triangles) forming three main clusters (*n* = 121 organoid lines from *n* = 23 AF samples). These clusters show colocalization with primary fetal tissue-derived control organoids (circles, *n* = 20) produced from lung (cyan), small intestine (purple), kidney (green), placenta (yellow), bladder (orange) and stomach (red) samples. **i**, scRNA-seq UMAP produced from representative AFOs from the three tissue identities. Epithelial cells are highlighted in orange, as identified by the SingleR cell-labeling package; KAFOs (1,467 cells, *n* = 5 patients), LAFOs (1,966 cells, *n* = 4 patients) and SiAFOs (1,576 cells, *n* = 2 patients) are shown. Source data

**Extended Data Fig. 2:**
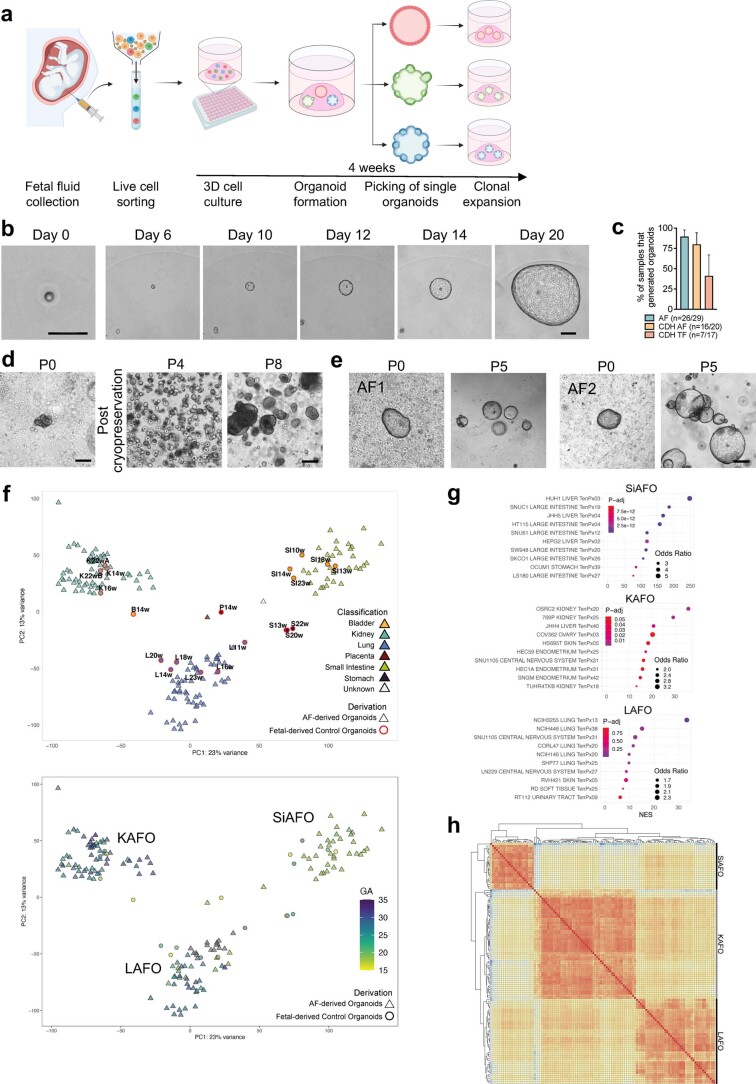
Derivation and characterization of AFO. **(a)** Schematic depicting the workflow for clonal AFO derivation. Image created and adapted in full licensed Biorender. **(b)** Phase-contrast images showing growth of clonal AFO from sorted AF epithelial cells cultured at a ratio of 5 cells in 3μL Matrigel droplet. **(c)** Percentage of samples that generated organoids at passage 0 (n=26/29 AF, n=16/20 CDH AF, n=7/17 TF samples, mean and 95% confidence interval [AF, 72.65, 97.81; CDH AF, 56.34, 94.27; TF, 18.44, 67.08]). **(d)** Phase-contrast images showing the recovery of AFO at passage 4 after cryopreservation and their expansion until passage 8 (Scale bar: 200 *μ*m). **(e)** Phase-contrast images depicting two additional AFO lines from independent patients (scale bar: 200 *μ*m). **(f)** PCA shows all sequenced AFO with the excluded unknown sample (top); PCA showing each organoid as its gestational age (bottom). **(g)** Top10 pathways from gene ontology analysis comparing each AFO identity to the other 2 AFO identities, (one-sided Fisher test, adjusted using Benjamini-Hochberg for multiple hypotheses). **(h)** Euclidean-clustered heatmap confirming the tissue-typing labeling of the AFO. Source data

To investigate formation of AFOs we seeded viable AF cells in Matrigel droplets and cultured them in an ad hoc*-*defined generic epithelial medium without tissue-specific signals (Supplementary Table 3). Individual AF cells began proliferating and self-organizing to form 3D organoids, visible within 2 weeks. To establish clonal lines, individual AFOs were picked, dissociated into single cells and replated (Fig. 2a,b and Extended Data Fig. 2a). Using this method, we derived 423 AFO lines from 42 AF samples (16–34 weeks gestational age (GA); Supplementary Tables 1 and 2). The clonal origin of AFOs was further confirmed by single-cell AFEpC culture (Extended Data Fig. 2b). AFOs showed multiple morphologies, expanded up to passage 20 and successfully cryopreserved, providing evidence of self-renewal and long-term culture (Fig. 2c,d and Extended Data Fig. 2b–e). Organoid formation was observed in 89.7% of AF samples, with a median formation efficiency of 0.011% (one organoid formed per 7.4 × 10^3^ cells; Fig. 2b and Extended Data Fig. 2c). We found no meaningful association between GA and AFO formation efficiency. We imaged two distinct organoid morphologies through X-ray phase-contrast computed tomography (PC-CT), confirming the applicability of the method for organoid characterization (Fig. 2e). Immunostaining confirmed cell proliferation (Ki67) and lack of apoptosis (cleaved caspase 3) within AFOs (Fig. 2f). We confirmed the AFO’s epithelial identity by staining for pan-epithelial markers (EpCAM, ECAD and pan-cytokeratin) and showing absence of the mesenchymal marker platelet-derived growth factor receptor α (PDGFRα). Notably, AFO’s epithelium is polarized as demonstrated by basolateral integrin β4 (ITGβ4), apical F-actin and zonula occludens-1 (ZO-1)-positive luminal tight junctions (Fig. 2g).

We then conducted bulk RNA sequencing to investigate the tissue identity of each clonal AFO line. As a control, we isolated tissue-derived human organoids from fetal small intestine (*n* = 5), stomach (*n* = 3), lung (*n* = 6), kidney (*n* = 4), bladder (*n* = 1) and placenta (*n* = 1; Fig. 1e). Unsupervised principal-component analysis (PCA) conducted on bulk RNA-seq data (*n* = 121 AFOs, 23 AF samples; Supplementary Table 2) showed three AFO clusters colocalizing with small intestinal, pulmonary and renal fetal tissue-derived control organoids (Fig. 2h and Extended Data Fig. 2f). No colocalization was observed with bladder or stomach organoids, whereas one AFO clustered with the placental control. Cluster-based Gene Ontology analysis corroborated the single tissue identity of each AFO clone, showing upregulation of pathways specific to the assigned tissues (Extended Data Fig. 2g). Annotation was further confirmed by Euclidean hierarchical clustering (Extended Data Fig. 2h). Finally, scRNA-seq conducted on AFOs from each cluster (small intestine *n* = 3, kidney *n* = 6 and lung *n* = 4; Supplementary Table 2) highlighted multiple epithelial cell clusters (Fig. 2i). Overall, this provides evidence that intestinal, renal and pulmonary AFEpCs can give rise to clonal AFO lines reflecting their respective tissue of origin.

### Characterization and maturation of SiAFOs

**Fig. 3:**
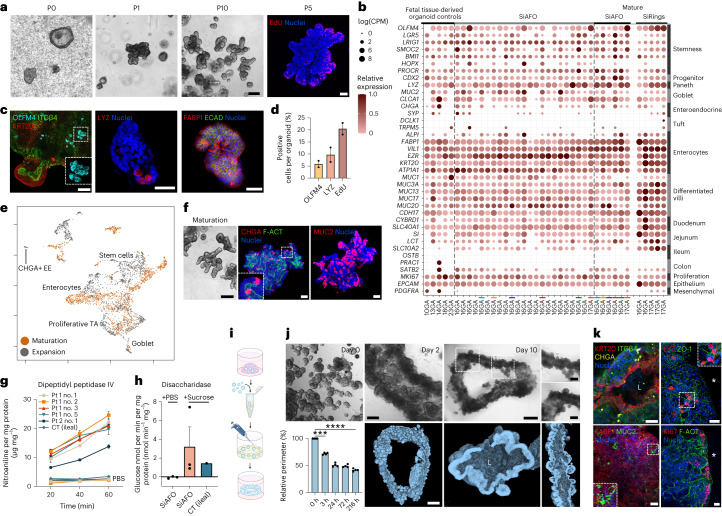
Characterization and maturation of small intestine AFO. **a**, Phase-contrast images depicting SiAFO expansion (scale bar, 200 μm). EdU assay showing proliferating cells localized at the crypt-like structure bases (scale bar, 50 μm). **b**, RNA-seq dot plot showing presence of small intestine markers in SiAFOs (*n* = 2 independent biological samples, *n* = 23 lines in expansion, *n* = 6 mature lines, *n* = 5 rings, *n* = 5 control fetal tissue-derived small intestinal organoids). **c**, Immunofluorescence for intestinal stem cell marker OLFM4, enterocyte marker KRT20 and ITGβ4. Paneth cells and enterocytes are highlighted by LYZ, FABP1 and ECAD staining (scale bars, 50 μm). **d**, Quantification of OLFM4, LYZ and EdU in SiAFOs (*n* = 2 independent biological samples; ≥4 organoids per sample; mean ± s.e.m.). **e**, Annotated scRNA-seq UMAP of representative SiAFOs in expansion (gray; 1,576 cells, *n* = 3 organoid lines) and maturation (orange; 1,666 cells, *n* = 3 organoid lines). **f**, Matured SiAFOs show budding morphology (scale bar, 200 μm). Immunofluorescent staining displays CHGA-positive enteroendocrine cells and MUC2-positive secretory cells. Counterstain with phalloidin (F-ACT) and Hoechst shows organoids’ lumen and nuclei, respectively (scale bars, 50 μm). **g**, Functional assessment of dipeptidyl peptidase IV activity (*n* = 2 independent biological samples; *n* = 5 clonal organoid lines at P6 and P10; *n* = 1 pediatric small intestinal ileal organoid control; mean ± s.e.m.). **h**, Functional evaluation of disaccharidase activity (*n* = 2 independent biological samples; *n* = 3 clonal organoid lines; *n* = 1 pediatric small intestinal ileal organoid control; mean ± s.e.m.). **i**,**j**, Schematic (created using BioRender) (**i**) and phase-contrast images (**j**, top) showing self-assembly and compaction of SiAFO ring (scale bars, 200 μm, 1 mm and 500 μm for insets; *n* = 8 rings from two AFs). Quantification of the ring’s relative perimeter over time (*n* = 4 independent experiments; mean ± s.e.m.; ***P = 0.002, ****P *<* 0.0001, one-way ANOVA multiple comparisons). MicroCT 3D reconstructions and cross-sections of a whole SiAFO ring depicting luminal structure (**j**, bottom) (scale bar, 50 μm). L, lumen. **k**, SiAFO rings display a lumen and KRT20-positive enterocytes, with ITGβ4 highlighting the basal side along with CHGA-positive enteroendocrine cells. The panel displays LYZ-positive cells, ZO-1-positive tight junctions, FABP1-positive enterocytes and MUC2 secretory cells in the SiAFO ring; proliferating Ki6-positive cells are observed in the crypt-like domain (*, external side) (scale bars, 50 μm). CT, control; PBS, phosphate-buffered saline. Source data

**Extended Data Fig. 3:**
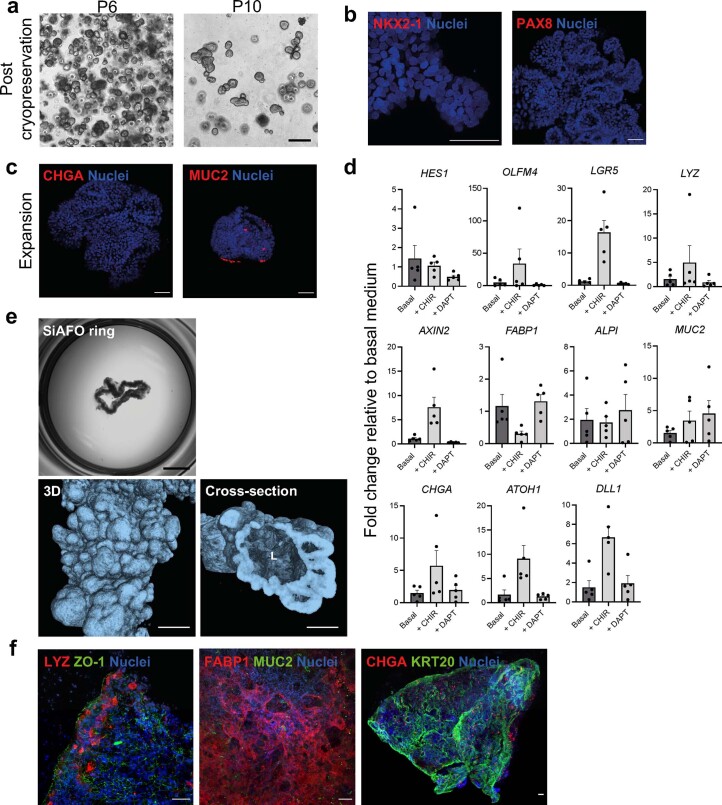
Characterization of SiAFO. **(a)** Phase-contrast images showing SiAFOs at passage 6 and their expansion after cryopreservation until passage 10 (scale bar: 200 *μ*m). **(b)** Whole-mount immunofluorescent staining showing the absence of NKX2-1 (lung) and PAX8 (kidney) in SiAFO (scale bar: 50 *μ*m). **(c)** Whole-mount immunofluorescent staining for Chromogranin A (CHGA) and Mucin 2 (MUC2) on SiAFOs in expansion medium (scale bar: 50 *μ*m). **(d)** RT–qPCR analysis of SiAFOs cultured in maturation medium without (Basal), with CHIR99021 (CHIR) or with DAPT (n=5 SiAFO clonal lines from n=2 patients; mean±SEM). **(e)** Stereoscopic image of an intestinal ring (scale bar: 1 mm) and microCT images showing the presence of budding (left) as well as luminal-like structures (L) (scale bars: 100 *μ*m). **(f)** 3D z-stacks of SiAFO rings showing tight junctions (ZO-1), Paneth cells (LYZ), enterocytes (FABP1 and KRT20), intestinal secretory cells (MUC2) and enteroendocrine cells (CHGA); nuclei counterstained with Hoechst (scale bars:50*μ*m). Source data

Small intestinal AFOs (SiAFOs) expanded consistently for more than ten passages, forming crypt-like structures (Fig. 3a and Extended Data Fig. 3a). Moreover, 5-ethynyl-2'-deoxyuridine (EdU) incorporation indicated cell proliferation at the SiAFO crypt base (Fig. 3a). Bulk RNA-seq of 23 SiAFOs showed expression of typical intestinal stem/progenitor (*LGR5*, *OLMF4*, *LRIG1* and *SMOC2*), Paneth (*LYZ*), goblet (*MUC2* and *CLCA1*), endocrine (*CHGA*) and enterocyte (*ALPI*, *FABP1*, *VIL1*, *EZR*, *KRT20* and *ATP1A1*) markers (Fig. 3b). Immunostaining for the stem cell marker olfactomedin 4 (OLFM4) and intestinal epithelial cytokeratin 20 (KRT20) confirmed the presence of a crypt–villus axis. SiAFO immunostaining showed markers of numerous intestinal cell types (Fig. 3c,d) such as Paneth cells (lysozyme (LYZ)) and enterocytes (fatty acid-binding protein 1 (FABP1)). Notably, SiAFOs lack lung (NKX2-1) and kidney (PAX8)-specific markers (Extended Data Fig. 3b). scRNA-seq confirmed the presence of tissue-specific cellular identities such as intestinal stem cells, proliferating transit amplifying, enterocytes, goblet and enteroendocrine cells (Fig. 3e).

We then performed a maturation assay culturing SiAFOs in intestinal-specific medium for 14 d. Upon maturation, SiAFOs displayed more prominent budding structures, resembling small intestinal crypt-like organization. Immunofluorescent (IF) staining showed chromogranin A (CHGA)-positive enteroendocrine cells and stronger presence of goblet cell marker mucin 2 (MUC2) compared to pre-maturation (Fig. 3f and Extended Data Fig. 3c). In addition, quantitative reverse transcription PCR (RT–qPCR) following DAPT treatment demonstrated downregulation of Notch target genes (*HES1* and *OLFM4)*, stem/progenitor cells, Paneth cells and Wnt target genes (*LGR5*, *LYZ* and *AXIN2*). While enterocyte and goblet cell markers (*FABP1*, *ALPI* and *MUC2*) were upregulated, the enteroendocrine marker *CHGA* was not. Notably, Notch inhibition did not drive increased expression of *ATOH1* nor its downstream target *DLL1* (Extended Data Fig. 3d).

To evaluate SiAFO functional capacity, we assessed the digestive activity of two small intestinal brush border enzymes, dipeptidyl peptidase IV (peptide hydrolysis; Fig. 3g) and disaccharidase (sucrose hydrolysis; Fig. 3h). Finally, to investigate the potential of SiAFOs for tissue engineering, we performed an intestinal ring formation assay (Fig. 3i). Three hours after seeding, SiAFOs started fusing; by day 2 a more complex tubular budding structure had formed with full ring maturation at day 10. MicroCT revealed SiAFO ring self-organization, forming a tube-like structure with lumen and buddings resembling intestinal architecture (Fig. 3j, Extended Data Fig. 3e and Supplementary Video 1). SiAFO rings manifested correct cell polarity (KRT20 and ITGβ4) and tight junctions (ZO-1), in addition to enterocytes (FABP1 and KRT20), enteroendocrine (CHGA), Paneth (LYZ) and goblet secretory cells (MUC2). Moreover, SiAFO rings maintained proliferation (Ki67) in the crypt-like portion (Fig. 3k, Extended Data Fig. 3f and Supplementary Video 2). The overall marker profile of SiAFO rings (bulk RNA-seq in Fig. 3b), revealed strong upregulation of genes typical of functionally differentiated intestinal cells, particularly of mucin secretory lineages, brush border enzymes and enterocytes.

### Characterization and differentiation of KAFOs

**Fig. 4:**
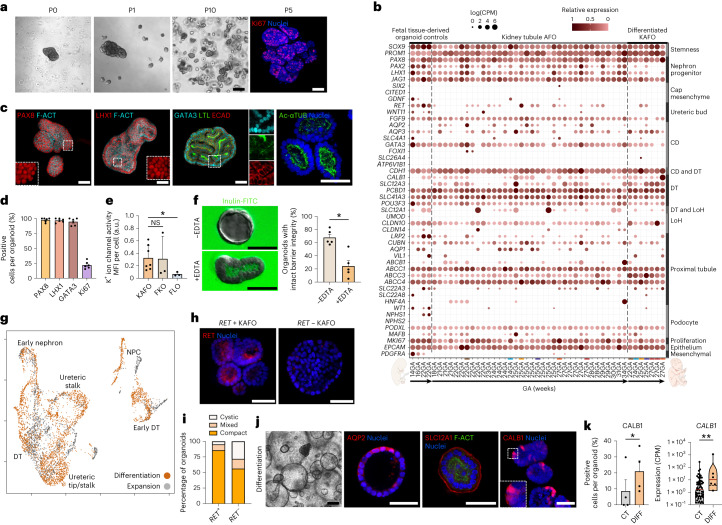
Characterization and differentiation of kidney tubule AFOs. **a**, Phase-contrast images showing long-term KAFO culture (scale bar, 200 μm). Immunofluorescent staining highlights proliferative marker Ki67 (scale bar, 50 μm). **b**, Bulk RNA-seq showing broad kidney markers’ presence in KAFOs (*n* = 19 independent biological samples, *n* = 37 lines in expansion, *n* = 7 lines in differentiation medium, *n* = 4 control FKOs). CD, collecting duct; DT, distal tubule; LoH, loop of Henle. **c**, Immunofluorescent staining shows presence of nephron progenitor markers PAX8 and LHX1 counterstained with phalloidin (F-ACT) and positivity for distal tubule/collecting duct marker GATA3, proximal tubule marker LTL, ECAD and Ac-αTUB apical cilia, further confirming the renal epithelial identity of KAFOs (scale bars, 50 μm). **d**, Quantification of renal markers PAX8, LHX1, and GATA3 in KAFOs (*n* = 6 independent biological samples, ≥4 organoids per sample; mean ± s.e.m.). **e**, Potassium ion channel assay performed on *n* = 7 KAFOs from independent biological samples, *n* = 3 FKOs, *n* = 3 FLOs as negative control (mean fluorescence intensity (MFI) was calculated; mean ± s.e.m., one-way ANOVA with multiple comparisons; *P = 0.0393). **f**, Images showing inulin assay results from untreated and EDTA-treated KAFOs (scale bars, 50 μm); percentage quantification of organoids with intact barrier integrity (no inulin-FITC uptake; *n* = 5 independent biological samples; mean ± s.e.m.; *P = 0.0121, two-tailed paired *t*-test). **g**, Annotated KAFO scRNA-seq UMAP in expansion (gray; 1,467 cells, *n* = 6 KAFO lines) and differentiation (orange; 3,559 cells, *n* = 3 KAFO lines). NPC, nephron progenitor cells. **h**, Immunofluorescent staining showing RET protein localization in *RET*^+^ and *RET*^−^ KAFOs (scale bars, 50 μm). **i**, Stacked bar chart representing proportion of organoids with compact, cystic, or mixed (compact/cystic) morphology in *RET*^+^ and *RET*^−^ KAFOs (*P = 0.0255, Spearman rank test). **j**, Phase-contrast and immunofluorescent (IF) images highlighting morphological changes, as well as the expression of the mature renal markers AQP2, SLC12A1 and CALB1 in KAFOs upon differentiation (scale bars, 200 μm phase-contrast and 50 μm IF). **k**, Left: quantification of CALB1-positive cells in KAFOs cultured in expansion (CT) and differentiation (DIFF) medium (*n* = 4 independent biological samples, mean ± s.e.m.; *P = 0.0357, two-tailed paired *t*-test). Right: *CALB1* gene expression in differentiated KAFOs (DIFF) compared to undifferentiated controls (CT) based on the RNA-seq plot presented in **b** (*n* ≥ 6 differentiated independent biological samples; mean ± s.e.m.; **P = 0.0029, unpaired *t*-test). Source data

**Extended Data Fig. 4:**
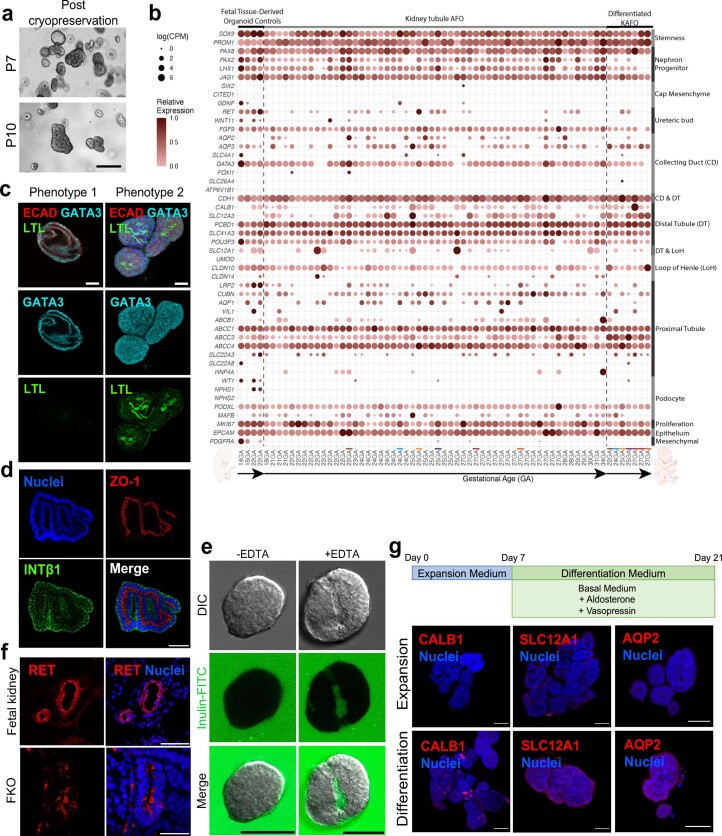
Characterization of KAFO. **(a)** Phase-contrast images showing the thawing of KAFOs after cryopreservation at passage 7 and their expansion until passage 10 (scale bar: 200 *μ*m). **(b)** Full set of KAFOs subjected to bulk RNA-seq. **(c)** Double positive GATA3 and LTL cells confirm a mixed tubular phenotype; in the left panel a different phenotype of KAFOs was observed with GATA3 positive and LTL negative cells (scale bar: 50 *μ*m). **(d)** Immunofluorescence showing presence of ZO-1-positive tight junctions in KAFOs (scale bar: 50 *μ*m). **(e)** Representative example of inulin-permeability assay (scale bar: 50 *μ*m). **(f)** Localization of RET protein signal in fetal kidney tissue slice and tissue-derived fetal kidney organoids (FKO; scale bar: 50 *μ*m). **(g)** Distal and collecting duct markers are absent or poorly present in KAFOs in expansion medium; differentiation induces expression of CALB1, SLC12A1 and AQP2 (n=4 independent biological samples); nuclei counterstained with Hoechst (scale bar: 50 *μ*m).

Kidney tubule AFOs (KAFOs) expanded long-term (up to passage 10), while maintaining proliferation (Ki67; Fig. 4a and Extended Data Fig. 4a) and mostly manifested a compact morphology with several lines showing a cystic structure. Bulk RNA-seq of 54 KAFOs (19 AF samples; 18–34 weeks GA) showed expression of canonical developmental renal epithelial and nephron progenitor genes (*PAX2*, *PAX8*, *LHX1* and *JAG1*), while lacking cap mesenchyme markers (*SIX2*, *CITED1* and *GDNF*). Moreover, we detected distal (*PCBD1*, *SLC41A3* and *POU3F3*) and proximal (*ABCC1*, *ABCC3*, *ABCC4* and *CUBN*) tubule gene expression. Collecting duct marker *GATA3* was expressed, while the loop of Henle marker *UMOD* was not. Podocyte markers (*WT1*, *NPHS1* and *NPHS2*) were not expressed in KAFOs except *PODXL* and *MAFB* (Fig. 4b and Extended Data Fig. 4b). Based on this, we concluded that KAFOs have a tubuloid-like phenotype and express markers belonging to different renal tubule segments, confirmed by protein expression of PAX8 and LHX1. KAFOs also displayed the segment-specific kidney tubule proteins GATA3 and ECAD (distal tubule/collecting duct). Conversely, we detected LTL, representing proximal tubule identity. Of note, KAFOs exhibited a mixed tubular phenotype, coexpressing GATA3 and LTL or presenting only GATA3. The presence of polarized tubular microvilli was validated by immunofluorescence for acetylated tubulin (Ac-αTUB) (Fig. 4c,d and Extended Data Fig. 4c). Additionally, KAFOs showed increased intracellular thallium fluorescence compared to fetal lung organoids (FLOs), comparable to control fetal kidney organoids (FKOs), indicating the presence of functional voltage-gated potassium channels (Fig. 4e). Moreover, KAFOs display functional epithelial tight junctions, with apical ZO-1 (Extended Data Fig. 4d) and intact barrier integrity with 67.9% of KAFOs impermeable to inulin-FITC diffusion, decreased to 24.4% upon ethylenediaminetetraacetic acid (EDTA) treatment (Fig. 4f and Extended Data Fig. 4e). Further scRNA-seq characterization conducted on KAFOs confirmed the presence of multiple renal tubule cells such as ureteric tip/stalk and distal tubule or early nephron and nephron progenitor cells (Fig. 4g). Notably, some KAFO lines (21 of 54) expressed ureteric bud marker *RET* (Fig. 4b and Extended Data Fig. 4b). RET protein staining on the luminal side presented comparably to fetal kidney sections and FKOs. (Fig. 4h and Extended Data Fig. 4f). In addition, 85.7% of *RET-*expressing KAFO lines exhibited compact morphology, this decreased to 54.5% in *RET*-negative lines, exhibiting more cystic or mixed morphology (Fig. 4i). Finally, we adapted a reported protocol to differentiate KAFOs toward a distal/collecting duct phenotype. After 14 d of vasopressin and arginine-aldosterone stimulation, KAFOs manifested markers of the collecting duct (AQP2) and distal tubules (SLC12A1 and CALB1), which were lower in expansion medium (Fig. 4j and Extended Data Fig. 4g). Additionally, differentiated KAFOs displayed more CALB1-positive cells upon immunostaining (21 ± 6.6%) and increased *CALB1* gene expression (Fig. 4k).

### Characterization and differentiation of LAFOs

**Fig. 5:**
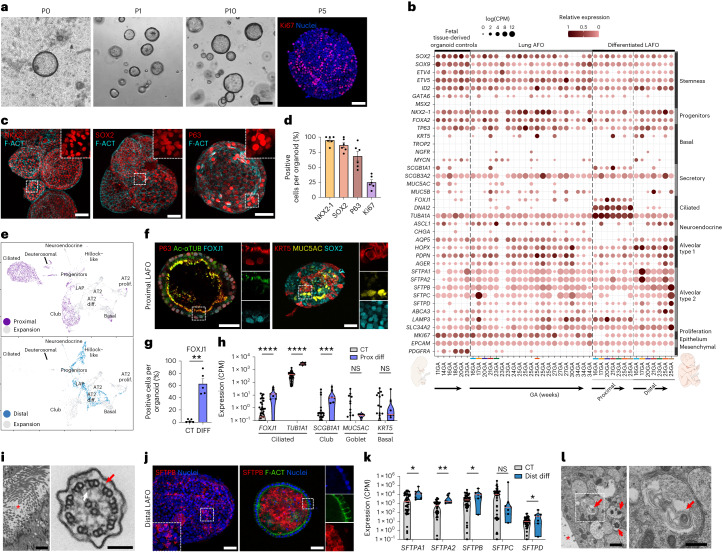
Characterization and differentiation of lung AFOs. **a**, Phase-contrast images depicting LAFO expansion (scale bar, 200 μm). IF staining highlights proliferative marker Ki67 (scale bar, 50 μm). **b**, Dot plot showing representative bulk RNA-seq gene expression in LAFOs (*n* = 12 independent biological samples, *n* = 21 undifferentiated LAFOs, *n* = 14 differentiated LAFOs, *n* = 6 control FLOs). **c**, IF staining highlighting the presence in LAFOs of lung stem/progenitor cell markers NKX2-1 and SOX2 together with P63 basal cells; counterstaining with phalloidin (F-ACT) (scale bars, 50 μm). **d**, IF quantification (*n* = 6 AF samples, ≥4 organoids per sample; mean ± s.e.m.). **e**, Annotated scRNA-seq UMAPs of LAFOs in expansion (gray; 1,966 cells, *n* = 4 organoid lines), proximal (top; 3,371 cells, *n* = 3 organoid lines) and distal differentiation (bottom; 1,351 cells, *n* = 3 organoid lines). **f**, IF staining on proximally differentiated LAFOs reveals polarized expression of ciliary protein Ac-αTUB, and ciliated cell marker FOXJ1. The panel also shows expression of basal cell markers P63, KRT5, presence of mucin 5AC goblet cells and maintenance of SOX2 progenitor cells (scale bars, 50 μm). **g**, Quantification of FOXJ1-positive cells within LAFOs in expansion (CT) versus proximal differentiation (DIFF) (*n* = 5 independent biological samples, ≥4 organoids per sample; mean ± s.e.m.; **P = 0.0016, two-tailed paired *t*-test). **h**, Violin plot showing gene expression of proximal airway markers *FOXJ1*, *TUBA1A*, *SCGB1A1*, *MUC5AC* and *KRT5* in proximal LAFOs (DIFF) compared to undifferentiated controls (CT) based on the RNA-seq plot presented in **b** (*n* ≥ 7 independent biological samples; median and quartiles; *SCGB1A1* ***P = 0.0003, ****P < 0.0001, Holm–Šídák multiple unpaired *t*-test). **i**, TEM images showing proximal LAFOs with cilia inside the lumen (asterisk) (scale bar, 2 μm); in cross-section, axonemes showing outer (red arrow) and inner (white arrow) dynein arms (scale bar, 100 nm). **j**, Distalized LAFOs showing surfactant-secreting cells (SFTPB) with granular and luminal secretion; Hoechst-counterstained nuclei (scale bars, 50 μm). **k**, Violin plot showing surfactant-related gene expression in distalized LAFOs (DIST DIFF) compared to control in expansion (CT) based on the RNA-seq plot presented in **b** (*n* ≥ 7 independent biological samples; median and quartiles; NS, non-significant; *SFTPA1* *P = 0.0107, *SFTPA2* **P = 0.0002, *SFTPB* *P = 0.0168, *SFTPD* *P = 0.0028, Holm–Šídák multiple unpaired *t*-test). **l**, Left: TEM of distalized LAFOs showing lumen (*) and cells containing lamellar bodies (red arrows). Right: magnification of lamellar body containing multi-lamellar membranes. Scale bars, 1 μm and 500 nm, respectively. Source data

**Extended Data Fig. 5:**
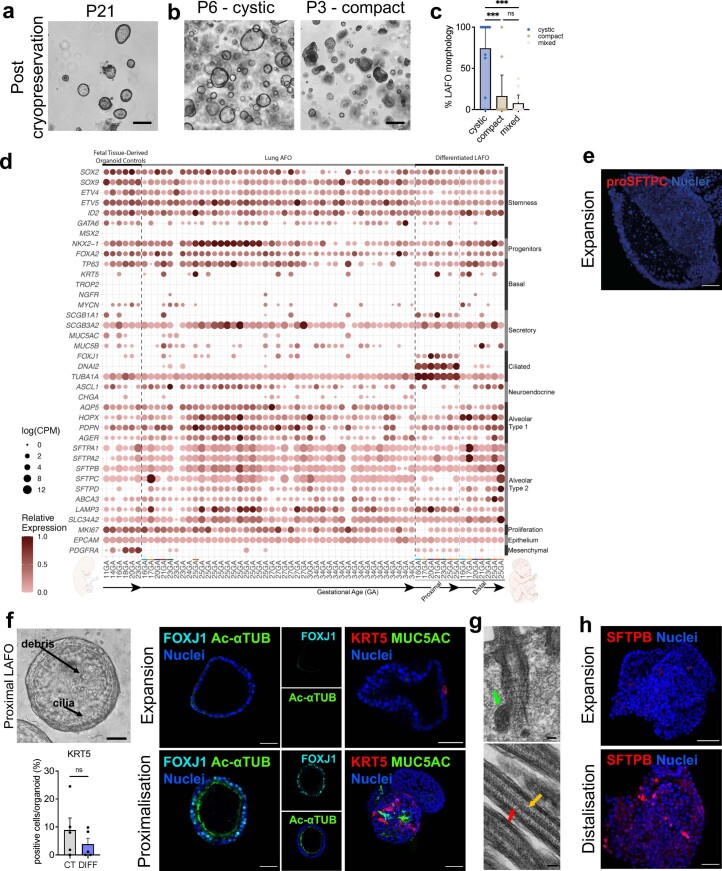
Characterization of LAFO. **(a)** Phase-contrast images showing LAFOs after cryopreservation expanded to passage 21 (Scale bar: 200 *μ*m). **(b)** Different LAFO lines’ morphological phenotypes (scale bars: 200 *μ*m), quantified in **(c)** (n=10 AF samples; mean with 95% CI, ***P<0.001, one-way ANOVA with multiple comparisons). **(d)** Full set of sequenced LAFOs. **(e)** LAFOs show absence of surfactant protein C in expansion (Scale bar: 50 *μ*m). **(f)** Airway markers (FOXJ1, ACATUB, MUC5AC, KRT5) are absent or poorly expressed at protein level in LAFOs cultured in expansion medium, but present in proximally differentiated LAFOs (n=5 independent biological samples, mean±SEM, ns=non-significant, two-tailed paired t-test) (Scale bar: 50 *μ*m). **(g)** TEM showing normal cilia rootlets with associated mitochondria (green arrow); longitudinal section of the axonemes confirms a normal central microtubule pair (yellow arrow) and radial spokes (red arrow) (scale bars: 100 nm). **(h)** Distalised LAFOs produce surfactant protein B vs. control in expansion; nuclei counterstained with Hoechst (scale bars: 50 *μ*m). Source data

Lungs are major cellular contributors to AF, continuously releasing fluid into the amniotic cavity. Consequently, lung AFOs (LAFOs) formed from the majority of our samples. We isolated, clonally expanded and sequenced 43 LAFO lines from 12 AF samples spanning 16–34 weeks GA. LAFOs were propagated up to 21 passages maintaining high proliferation, confirmed by Ki67 staining (Fig. 5a and Extended Data Fig. 5a). LAFOs can have cystic or compact morphology (Extended Data Fig. 5b,c). Bulk RNA-seq indicated expression of multiple pulmonary markers in 43 LAFOs, manifesting stem/progenitor cell markers (*NKX2-1*, *FOXA2*, *SOX2*, *SOX9*, *TP63* and *GATA6*), alveolar type 1 (*HOPX*, *PDPN*, *AGER* and *AQP5*) and alveolar type 2 cell-related genes (*SFTPA1*, *SFTPA2*, *SFTPB*, *SFTPC*, *SFTPD*, *ABCA3* and *LAMP3*). Mature basal cell markers *KRT5*, *TROP2* and *NGFR* were rarely detected in LAFOs as well as the ciliated cell transcription factor *FOXJ1*, which showed sporadic low expression in the expansion medium. Furthermore, the early secretory cell marker *SCGB3A2* was highly expressed compared to the absent mature club cell marker *SCGB1A1*. Secretory goblet cell marker *MUC5AC* was expressed in tissue-derived control FLOs, but several LAFO lines showed low/negligible expression. Last, while the neuroendocrine cell marker *ASCL1* was expressed in almost all LAFOs, only a small number showed expression of *CHGA* (Fig. 5b and Extended Data Fig. 5d). Immunostaining demonstrated homogenous protein expression of the stem cell markers NKX2-1 and SOX2 and basal cell marker P63 (Fig. 5c,d). Pro-surfactant protein C (proSFTPC) was absent (Extended Data Fig. 5e).

We then investigated the proximal and distal differentiation of LAFOs. scRNA-seq indicated successful proximal airway differentiation with newly formed specialized cell clusters such as ciliated and deuterosomal cells, associated with an increase in secretory cells (Fig. 5e; top UMAP). Notably, we also observed polarized epithelium with motile cilia on LAFO luminal surfaces (Supplementary Video 3). Immunofluorescent staining confirmed the presence of luminal Ac-αTUB cilia on proximally differentiated LAFOs, which was further corroborated by ciliated epithelia marker FOXJ1 nuclear expression (Fig. 5f). In addition, the appearance of keratin 5 (KRT5; mature basal cells) and secretory marker mucin (MUC5AC) concomitantly with the maintenance of SOX2, corroborated proximal lung differentiation (Fig. 5f and Extended Data Fig. 5f). FOXJ1-positive cells (62.8 ± 8.2%) increased in the differentiated LAFOs compared to controls in expansion, whereas the number of KRT5-positive cells (3.8 ± 2.2%) did not (Fig. 5g and Extended Data Fig. 5f). Moreover, proximal LAFOs showed increased gene expression of airway markers such *FOXJ1, TUBA1A* and *SCGB1A1*, when compared to undifferentiated controls (Fig. 5h). Cilia were analyzed in detail by transmission electron microscopy (TEM), displaying normal rootlets with associated mitochondria. The ciliary axonemes also displayed normal structures with radial spokes and a normal central microtubule pair, outer and inner dynein arms (Fig. 5i and Extended Data Fig. 5g). When directed toward a distal phenotype, LAFOs increased protein expression of the AT2 marker SFTPB, displaying different cellular localization in independent LAFO lines. Some presented within intracellular granules, others as luminal surfactant accumulation, possibly indicating a more mature state (Fig. 5j and Extended Data Fig. 5h), alongside increased expression of *SFTPA*1, *SFTPA2*, *SFTPB* and *SFTPD* (Fig. 5k). Notably, scRNA-seq revealed that distally differentiated LAFOs showed increased *SCGB3A2*^*+*^/*SFTPB*^*+*^ lower airway progenitor cells, emergence of a Hillock-like cluster and reduction of club cells (Fig. 5e; bottom UMAP). Finally, ultrastructural analysis revealed that distal LAFOs contain lamellar bodies with a normal structure, typical of surfactant-secreting cells (Fig. 5l). Overall, this indicates progression of LAFOs toward more mature lung phenotypes.

### Characterization of AF and TF organoids from CDH fetuses

**Fig. 6:**
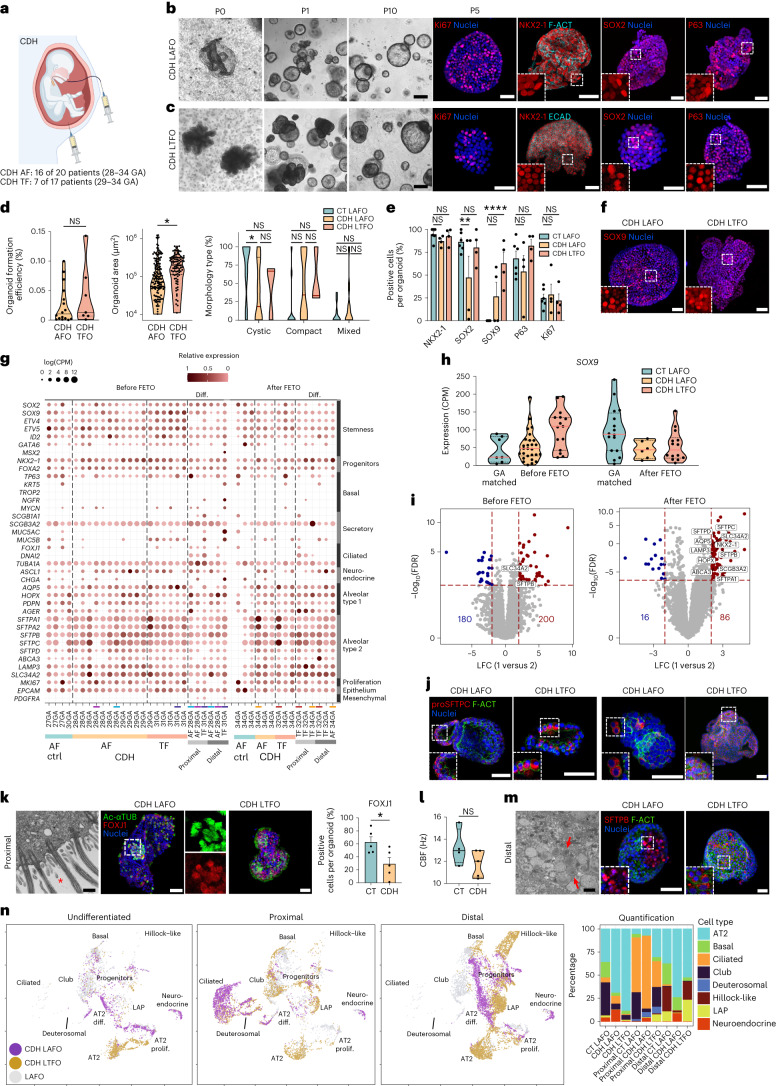
Generation, differentiation and characterization of LAFOs and LTFOs from CDH pregnancies. **a**, Schematic of AF/TF sampling from CDH pregnancies. **b**,**c**, Phase-contrast images depicting CDH LAFOs (**b**) and LTFOs (**c**) P0–P10 (scale bars, 200 μm). IF staining panel highlights proliferative marker Ki67, lung stem/progenitor markers NKX2-1/SOX2 and basal cell marker P63 in CDH organoids (scale bars, 50 μm); nuclei counterstained with Hoechst. **d**, Organoid formation efficiency (organoids per live cells, *n* = 16 CDH AF and *n* = 7 CDH TF independent samples; median and quartiles) and area of CDH AFOs versus CDH TFOs at isolation (*n* ≥ 91 organoids; median and quartiles; *P = 0.00482, unpaired *t*-test). Bar graph displays CDH LAFO/LTFO morphologies versus controls (cystic, compact, mixed; *n* ≥ 3 independent biological samples; median and quartiles; NS, non-significant; *P = 0.0477, two-way ANOVA with multiple comparisons). **e**, Immunofluorescent staining quantification (*n* = 7 CT AF, *n* = 4 CDH AF, *n* = 4 CDH TF independent samples, ≥4 organoids per sample; mean ± s.e.m.; NS, non-significant; **P = 0.0076, ****P < 0.0001, two-way ANOVA with multiple comparisons). **f**, IF staining showing SOX9 in CDH LAFOs/LTFOs (scale bars, 50 μm). **g**, Dot plot of lung-related markers in CDH TFOs (29–34 GA, four patients) and AFOs (28–34 GA, eight patients) alongside GA-matched control LAFOs (27–34 GA, three patients). **h**, Violin plots showing *SOX9* expression before and after FETO in CDH LAFOs/LTFOs compared to GA-matched control LAFOs (median and quartiles). **i**, Volcano plots showing DEGs between CDH organoids and GA-matched controls before and after FETO. Significant (*P* < 0.01) lung-associated markers are labeled. The blue dots represent the statistically significant downregulated genes, and the red dots represent the statistically significant upregulated genes. LFC, log fold change. **j**, Immunofluorescent staining showing pro-surfactant protein C (proSFTPC) in CDH organoids before (left) and after (right) FETO (scale bars, 50 μm). **k**, TEM image shows proximal CDH LAFOs exhibiting cilia (*), confirmed with IF staining for Ac-αTUB and ciliary transcription factor FOXJ1; nuclei counterstained with Hoechst (scale bars, 500 nm TEM, 50 μm IF). FOXJ1 quantification is presented in the bar graph (*n* = 5 CDH and *n* = 5 CT independent biological samples, mean ± s.e.m. *P = 0.0318 two-tailed unpaired *t*-test). **l**, Quantification of CBF (Hz; *n* ≥ 5 videos per organoid line, *n* = 5 CDH organoid lines, *n* = 4 non-CDH control LAFO lines; median and quartiles; NS, non-significant, two-tailed unpaired *t*-test). **m**, TEM imaging showing presence of lamellar bodies (arrows) in distalized CDH organoids (scale bar, 1 μm). IF staining shows surfactant protein B (SFTPB) in distalized CDH LAFOs/LTFOs; nuclei counterstained with Hoechst (scale bars, 50 μm). **n**, Annotated scRNA-seq UMAPs of CDH LAFOs/LTFOs in expansion (left; 1,877 cells, *n* = 6 organoid lines), proximal (middle left; 3,843 cells, *n* = 5 organoid lines) and distal differentiation (middle right; 6,090 cells, *n* = 5 organoid lines). Stacked bar plot of cell types between organoid identities (%) is shown (right). Source data

**Extended Data Fig. 6:**
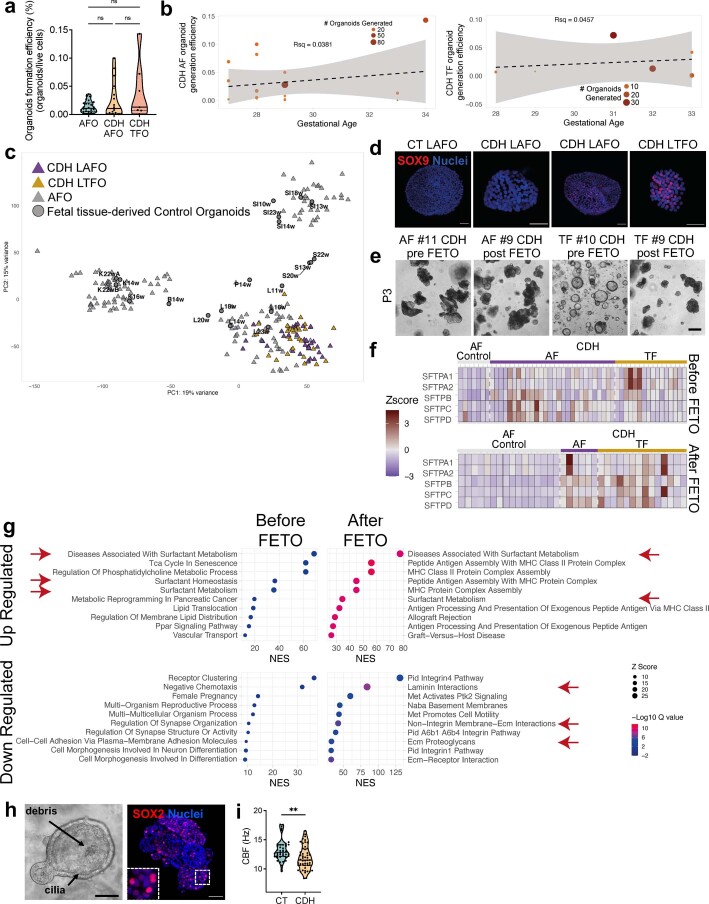
Derivation and characterization of CDH organoids. **(a)** Comparison of organoid formation efficiency between non-CDH and CDH fluids at P0 (n=26 non-CDH AFs; n=16 CDH AFs; n=7 CDH TFs; median and quartiles). **(b)** Organoid formation efficiencies at various gestational age for CDH AF and TF. **(c)** PCA showing CDH organoids alongside all the AFO clusters. **(d)** Representative CDH organoid lines stained for SOX9 (scale bars: 50 *μ*m). **(e)** Derivation of CDH AFO and TFO from different patients before (left) and after (right) FETO surgery (scale bar: 200 *μ*m). **(f)** Heatmap showing comparison of lung surfactant genes expression between CDH organoids before and after FETO. **(g)** GO analysis of CDH LAFO and LTFO before and after FETO, when compared to GA-matched control LAFOs; red arrows highlight surfactant and matrix remodeling pathways. **(h)** Phase-contrast image of Proximal CDH LAFOs showing cilia and mucus/debris within the lumen. Immunofluorescence image showing Proximal CDH LAFOs expressing SOX2 (scale bars: 50 *μ*m). **(i)** Ciliary beating frequency analysis of CDH organoids (n=49 organoids from 5 lines of 3 independent patients) vs CT LAFOs (n=40 organoids from 4 lines of 4 independent patients; **P=0.0014 unpaired t-test). Source data

**Extended Data Fig. 7:**
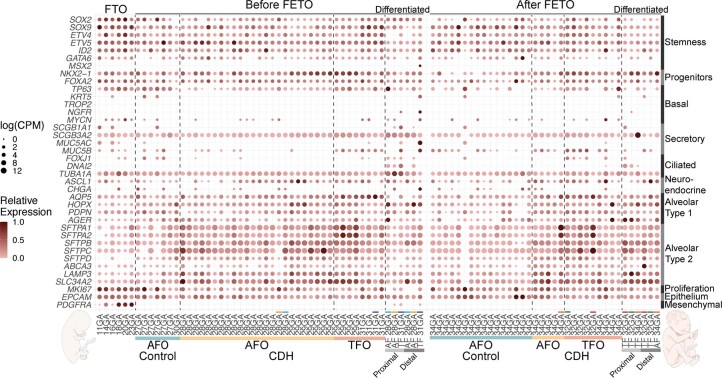
Dot plot showing expression of lung genes in CDH LAFO/LTFO. Expression of a selection of lung genes associated with different lung cell types (log CPM). Relative expression within each gene is shown by color. All CDH organoids generated, LAFO and LTFO are shown, separated by before and after FETO. Proximal and Distal differentiated CDH organoids also shown, with colored bars used to pair differentiated organoid with the matching undifferentiated organoid line. They are shown alongside GA-matched LAFO controls and tissue-derived fetal organoid controls (not age-matched).

**Extended Data Fig. 8:**
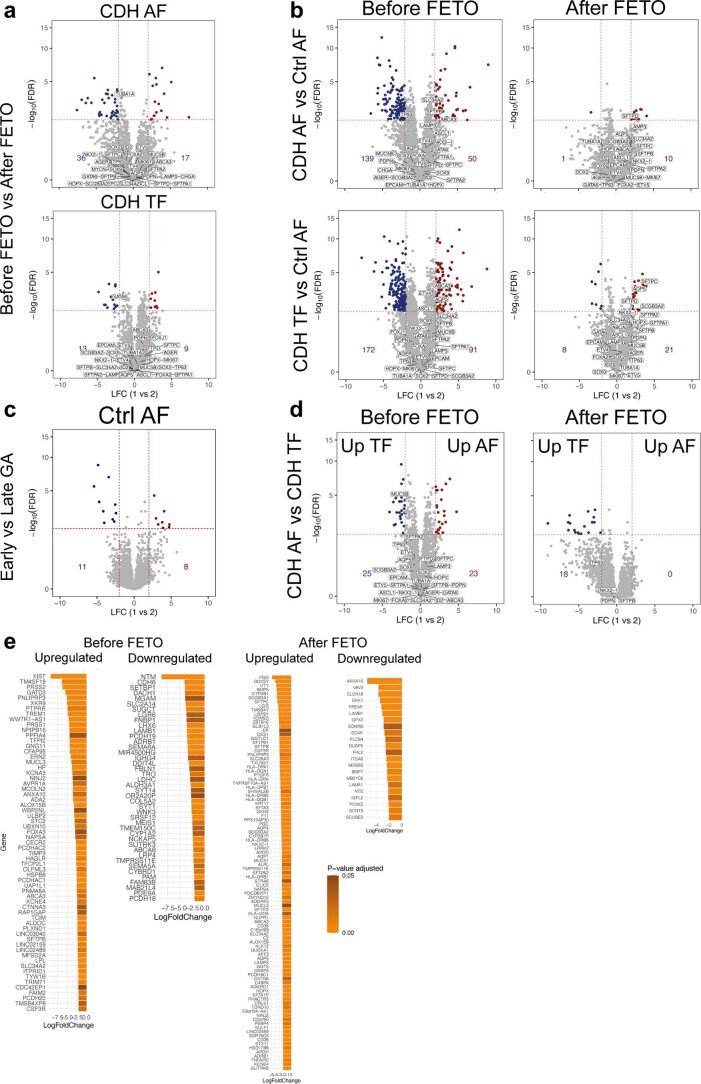
Additional CDH organoid gene expression analyses. **(a)** Volcano plots showing significant DEGs (p<0.05, |LFC| > 2). Lung-relevant genes are highlighted. Comparison of organoids generated from AF or TF sampled after FETO with those before FETO. **(b)** Comparison of LAFO and LTFO from before and after FETO with GA-matched control LAFOs. **(c)** Comparison of CT LAFO between samples GA-matched to before and after FETO **(d)** Comparison of LAFO with LTFO, before and after FETO. **(e)** DEG gene list generated from comparing CDH LAFO to controls, before and after FETO.

CDH is a rare congenital malformation where the diaphragm fails to close, with herniation of the abdominal organs into the chest (OMIM 142340). Consequently, fetal lungs are mechanically compressed, limiting growth of both respiratory and vascular compartments^35^. To test our platform for disease modeling, we derived lung organoids from both AF and TF of fetuses with severe/moderate CDH-related lung hypoplasia (Fig. 6a and Supplementary Table 1). Fluids were obtained at FETOs, from patients not receiving steroids^3,4^. AFOs were derived as above; however, due to the low TF sample volume (1–3 ml) and high cell viability (60–75%) we omitted sorting to preserve cell numbers. Similar to control (CT) LAFOs (Fig. 5), we successfully generated CDH AFOs from 16 AF samples (16 of 20; 80%) and CDH TF organoids (TFOs) from 7 TF samples (7 of 17; 41.2%; Fig. 6a and Extended Data Figs. 2a and 6a,b). CDH LAFOs and lung TFOs (LTFOs) expanded up to passage 10, manifesting morphology consistent with CT LAFOs (Fig. 6b–d). PCA confirmed that all TF-derived organoids had lung identity and demonstrated clustering of CDH LAFOs/LTFOs with an associated shift from CT LAFOs (Extended Data Fig. 6c). CDH organoids expressed NKX2-1, SOX2 and P63 lung markers, along with Ki67 (Fig. 6b,c,e). Notably, CDH organoids showed higher SOX9 immunofluorescence positivity than CT LAFOs (Fig. 6e,f and Extended Data Fig. 6d), suggesting a more prominent stem/progenitor identity^36^. CDH LAFOs/LTFOs were generated from samples taken at the two FETO-related interventions: (1) occlusive endotracheal balloon insertion (28–31 weeks GA); and (2) balloon removal (32–34 weeks GA; Extended Data Fig. 6e). Paired analysis was not possible due to limited sample availability and therefore pooled analysis was performed. Our RNA-seq data (*n* = 30 CDH LAFOs from eight patients, *n* = 23 CDH LTFOs from four patients) highlighted expression of lung epithelial stem/progenitor markers at levels similar to GA-matched CT LAFOs (Fig. 6g and Extended Data Fig. 7). *SOX9* expression was remarkably downregulated in CDH organoids generated post-FETO, consistent with increased tissue maturation (Fig. 6h). Comparative analysis between CDH and GA-matched control LAFOs showed a reduction in differentially expressed genes (DEGs) (*P* < 0.01, logfold change (FC) > 2) between organoids generated from samples before (380) and after (102) FETOs (Fig. 6i, with further comparisons shown in Extended Data Fig. 8). Gene Ontology analysis identified upregulated pathways related to surfactant production and metabolism in CDH organoids, upregulation of phosphatidylcholine metabolism, and downregulation of pathways related to laminin interaction, integrin/ECM interaction and ECM proteoglycans (*P* < 0.05, logFC > 1), more evident in post-FETO organoids (Extended Data Fig. 6f,g). SFTPC expression was also validated through immunofluorescent staining (Fig. 6j).

We then differentiated CDH LAFOs/LTFOs. Similar to controls, proximal CDH LAFOs showed cilia via TEM, acetylated α-tubulin (Ac-αTUB), FOXJ1 and SOX2 expression (Fig. 6k and Extended Data Fig. 6h). The ciliary beating frequency (CBF) for proximal CT and CDH LAFOs was shown to be within the normal physiological range using a high-speed camera (Fig. 6l and Supplementary Video 4). Finally, when subjected to distal differentiation, CDH organoids showed lamellar bodies and expressed SFTPB (Fig. 6m). scRNA-seq found substantial differences in cellular composition between differentiated and undifferentiated CDH LAFOs, compared to control LAFOs (Fig. 6n). CDH LAFOs/LTFOs showed decreased basal and club cell populations, consistent with CDH models^33^. We detected an increased percentage of pulmonary neuroendocrine cells in CDH LAFOs in expansion and differentiation, consistent with human CDH tissue specimens and animal models^37,38^. Notably, we revealed increased representation of AT2 cells in CDH LAFOs/LTFOs in conjunction with further differences in proximally and distally differentiated CDH organoids. CDH LAFOs did not display the expected increase in club cells following proximal differentiation but showed an increased deuterosomal cell fraction. Last, distalized CT LAFOs displayed an appearance of Hillock cells and lower airway progenitor cells, absent in distal CDH LAFOs. Of note, and in contrast, distalized CDH LTFOs displayed fewer basal cells (Fig. 6n).

## Discussion

This work demonstrates that the AF contains tissue-specific fetal epithelial progenitor cells originating from various developing organs. We show that, under defined culture conditions, these cells form epithelial organoids resembling their tissues of origin (small intestine, kidney and lung). Finally, we provide evidence that lung organoids derived from AF and TF of fetuses affected by CDH exhibit features of the disease.

Personalized therapeutic modeling of congenital conditions must be implemented prenatally. Here, we demonstrate derivation of autologous primary fetal organoids from AF and TF, sampled for clinical purposes while allowing continuation of pregnancy. The AFO technology uses widely available samples, requiring minimal manipulation and applying established culture techniques. Fluid sampling to AFO characterization and expansion is implemented within 4–6 weeks, a timeline relevant to prenatal intervention, counseling and therapy, with great advantages over iPS cell-dependent methods, requiring 5–9 months to produce organoids^9,10,39^.

For this work we compiled a single-cell atlas of unperturbed human AF. AF cells are known to be mostly epithelial, with their origin often ascribed to skin, kidneys and fetal membranes^40^. Initial AFEpC automatic annotation and reference integration attempts were unsuccessful. The AF has a nonspecific ambient RNA signature and contains cells that lack adhesion and are exposed to nutrients/gas imbalances. Moreover, currently available fetal atlases only cover earlier gestational stages and the cells analyzed are captured within their native tissue environment^41–45^ (Extended Data Fig. 1e). Our map expands on the heterogeneity of the human AFEpC, showing these to exhibit multiple tissue origins being therefore distinct from previously reported placental-derived amniotic epithelial cells^46,47^. Within AFEpCs we identified gastrointestinal, renal and pulmonary epithelial stem/progenitor cells. In culture, these lineage-committed progenitors generated clonal organoids capable of long-term expansion. Remarkably, upon maturation, SiAFOs, KAFOs and LAFOs acquired further tissue-specific differentiation hallmarks.

Derivation of SiAFOs was rare, with success in only two AF samples (16–17 weeks GA, one obtained from a termination of pregnancy). The occurrence of AFEpCs with intestinal progenitor features is interesting, as after the breakdown of the anal membrane (12 weeks GA), sphincters are expected to retain the intestinal content^48^; however, colonocytes are reported in second trimester AF^49^ suggesting possible release of gastrointestinal cells later in pregnancy. Thus, SiAFOs could find therapeutic use, for example where prenatal diagnosis of short-bowel syndrome is made. Notably, matured SiAFOs acquired further tissue-derived small intestinal organoid hallmarks^50^.

KAFOs were easily derived across all gestational ages, likely due to excretion from developing kidneys^29^. KAFOs recapitulated tubuloid identity^14^ through expression of nephron progenitor and tubule markers. Upon differentiation, KAFOs upregulated functional renal proteins such as AQP2, SLC12A1 and CALB1.

LAFO derivation was frequent, due to continuous provision of lung progenitors to AF via regular circulation of fluid through fetal lungs^51^, interrupted during FETO, possibly explaining the differences between CDH LAFOs and CDH LTFOs^52^. Current CDH patient stratification relies on simple and reliable prenatal imaging parameters^53^. FETO is of great survival benefit to the most severely affected patients, but better predictive functional biomarkers are needed as 60% of treated fetuses do not survive to hospital discharge^54^. For instance, previous work on TF showed a different miRNA signature (miR-200) in patients who were responsive to the intervention; however, we could not assess this in our dataset due to transforming growth factor β and BMP inhibition interference^55^. We showed that CDH LAFOs/LTFOs manifest altered expression of surfactant protein genes. Consistently, AT2/AT1 increase was previously suggested as a hallmark of hypoplastic and CDH lungs^56,57^. Our RNA-seq analyses demonstrated increased *AT2* gene expression in CDH organoid cells compared to GA-matched controls, suggesting that these manifest some disease features, supporting their potential use in prenatal regenerative medicine. Future investigations will determine whether there is correlation between alterations in CDH LAFOs/LTFOs and clinical outcomes of the fluid donors. If successful, AFOs could become a complementary prenatal prognostic tool. It should be noted that AF sampling after FETO is conducted before balloon removal, therefore CDH LAFOs may not reliably reflect changes in the fetal lung in response to FETO. CDH LTFOs that are sampled from the occluded trachea will do so^58^. Of note, although TF sampling at the time of FETO is less widely applicable, this gives access to pure fetal lung/airway cells that produce only lung organoids.

Among 239 organoids sequenced, we found only one to have an unknown phenotype (Extended Data Fig. 2e), ascribed to a non-clonality at the picking stage. For future endeavors, optimizing AFO derivation could involve cost-effective methods such as PCR and image-based characterization, rather than RNA sequencing. Disease modeling requires a larger CDH patient cohort for platform validation to finely model the disease and test treatments. Finally, the present system is limited to the epithelial compartment and cannot model complex conditions involving, for example, mesenchymal and vascular compartments. This might be overcome by culturing organoids with mesenchymal and endothelial cells. Notably, mesenchymal and hematopoietic cells can be isolated from AF^30,59^, and direct reprogramming of AF cells to the endothelial lineage has been reported^60^. Hence, AF can provide different cell lineages to generate more-complex prenatal models. Future work will also focus on deriving organoids for each AF-exposed tissue. As an example, we identified one AFO with placental identity, suggesting the possible presence of additional organoid-forming AFEpCs.

In conclusion, we report derivation of fetal epithelial organoids of different tissues from fetal fluids, through minimally invasive sampling. This is achieved from continuing pregnancies, within a GA window beyond the one currently accessible from fetal tissue obtained from termination of pregnancies. Small intestine, kidney tubule and lung AFOs are expandable and can be functionally matured with great potential for regenerative medicine and personalized disease modeling.

## Methods

### Ethics and informed consent

Fetal fluid samples were collected from all participants after written informed consent in compliance with the Declaration of Helsinki. Ethical approval was given by the NHS Health Research Authority, in accordance with the Governance Arrangements for Research Ethics Committees and complied fully with the standard operating procedures for research ethics committees in the UK. Fetal fluid samples were collected from the University College London Hospital (UCLH) Fetal Maternal Unit (FMU) under the London Bloomsbury Research Ethics Committee (REC 14/LO/0863 IRAS ID 133888) in compliance with UK national guidelines (Review of the Guidance on the Research Use of Fetuses and Fetal Material, 1989, Cm762). Fetal fluid samples were also collected and used at UZ Leuven (ethics committee no. S53548). Control fetal tissue samples were sourced via the Joint Medical Research Council (MRC)/Wellcome Trust Human Developmental Biology Resource under informed ethical consent from all donors, undertaken with Research Tissue Bank ethical approval (project 200478: UCL REC 18/LO/0822, IRAS ID 244325; Newcastle REC 18/NE/0290, IRAS ID 250012). Pediatric intestinal tissue sample was obtained upon informed consent and ethical approval for the use of human tissue obtained from the East of England, Cambridge Central Research Ethics Committee (REC 18/EE/1050). Details on recruitment and ethics oversight are also provided in the Reporting Summary.

### AF collection and isolation of the viable cell fraction

AF samples (amniocenteses and amniodrainages) were collected from UCLH FMU and UZ Leuven as part of standard patient clinical care. After collection, fluids were stored at 4 °C until processing. AF samples were passed through 70-μm and 40-μm cell strainers and transferred to 50-ml tubes before being centrifuged at 300*g* for 10 min at 4 °C. Supernatant was discarded, pellet resuspended in 5–10 ml FACS blocking buffer containing 1% FBS and 0.5 mM EDTA in PBS and transferred to FACS tubes. Cells were incubated with 5 μg ml^−1^ Hoechst (Sigma-Aldrich, 33342) for 40 min at 37 °C and then counterstained with 2 μg ml^−1^ PI (Sigma-Aldrich, P4170) for 5 min at room temperature. Viable cells were sorted using a FACSAria III (BD), unselected for side and forward scatter, but gated for Hoechst^+^ and PI^−^. Viability was confirmed through a Live/Dead Acridine Orange/PI fluorescent Luna cell counter.

### Derivation and culture of human AFOs

Viable AF cells were resuspended in cold Matrigel (Corning, 354230) and plated at a density of 6 × 10^4^ live cells per 30 μl droplet onto a pre-warmed 24-well plate. Cells were cultured in an ad hoc chemically defined generic medium (AFO expansion medium; Supplementary Table 3) supplemented with Rho-kinase inhibitor (ROCKi; Tocris) and Primocin (Invivogen, NC9141851) for the first 3 d of culture. To establish clonal organoid lines, single organoids formed at P0 (day 14–20) were manually picked under the microscope to be clonally expanded. Each individual organoid was assigned an ID line and transferred to a 0.5-ml tube pre-coated with 1% BSA (Sigma-Aldrich). Organoids were resuspended in TrypLE (Thermo, 12605010) and incubated for 5 min at 37 °C. After digestion, organoids were disaggregated by pipetting and an additional 400 μl ice-cold Advanced DMEM/F12 supplemented with Glutamax, P/S and HEPES (ADMEM+++) were added. Organoids were precipitated with a minicentrifuge for 2 min and a second washing passage with ADMEM+++ was repeated. After centrifugation, the pellet was resuspended in 20 μl cold Matrigel (Corning, 354230) and plated in a pre-warmed 48-well plate. The plate was incubated for 20 min at 37 °C and AFO expansion medium was added with ROCKi and Primocin (Invivogen, NC9141851) for the first 3 d (Supplementary Table 3). Medium was replaced every 3–4 d. After approximately 10–14 d, grown organoids were passaged as described below.

### Derivation and culture of human fetal LTFOs

Euploid fetal TFs were collected during procedures of FETO carried out at the UCLH or UZ Leuven (ethical approval REC 14/LO/0863 IRAS 133888 and ethics committee number S53548, respectively), kept refrigerated and processed within 24–48 h. We collected TF samples before the insertion of the balloon (pre-FETO) and after its removal (post-FETO). Due to the nature of the TF samples, mostly small (1–3 ml) and containing a majority of living cells, FACS sorting was not performed as it was not deemed necessary. TFs were transferred into 15-ml tubes on ice, washed with ice-cold ADMEM+++ and centrifuged at 300*g* for 5 min at 4 °C. Supernatant was discarded and cells were resuspended in 1 ml ADMEM+++. Cells were counted with Trypan blue to normalize and exclude dead cells and then plated in Matrigel (Corning, 354230) droplets as described for the AF cells above. Plates were incubated for 20 min at 37 °C and human fetal lung organoid medium (Supplementary Table 3) supplemented with ROCKi and Primocin (Invivogen, NC9141851) was added. The medium was changed every 3 d. LTFOs were clonally expanded with the same methodology described above for the AFOs and passaged as described below.

### Passaging of organoids

Depending on number and size, organoids were passaged for expansion into a 24 or 12-well plate after clonal picking. Afterwards organoids were usually split 1:2 to 1:3 every 10–14 d of culture. For passaging, the medium was aspirated and ice-cold ADMEM+++ was added to each well. Matrigel droplets were disrupted and collected into a 15-ml tube on ice. Organoids were washed with 10 ml cold ADMEM+++ and centrifuged at 300*g* for 5 min at 4 °C. Large and cystic organoids were resuspended in 1 ml ADMEM+++ and mechanically disaggregated using a P1000 pipette. If small, organoids were instead disrupted enzymatically as follows. The medium was aspirated and organoid pellets were resuspended in 300 μl TrypLE. After incubation for 3–7 min at 37 °C, organoids were pipetted with P200 to break them down into single cells. Cold ADMEM+++ was added up to 10 ml and the sample was centrifuged at 300*g* for 5 min at 4 °C. The supernatant was discarded and the cell pellet was resuspended in Matrigel (Corning, 354230) and plated. The plate was incubated for 20 min at 37 °C to allow the Matrigel to solidify, upon which AFO expansion culture medium was added with ROCKi. The medium was changed every 3 d.

### Fetal tissue collection and generation of a control primary fetal organoids library

Control fetal tissue organoid derivation was conducted as follows:

#### Human fetal tissue-derived small intestinal organoids

Fetal small intestines were processed as previously described^61^. The tissue was washed with PBS, cleared of any mesenteric tissue and fat, then cut longitudinally. The villi were scratched away using a glass coverslip. The remaining tissue was cut into 2–3-mm pieces, washed vigorously and incubated in 2 mM EDTA in PBS for 30 min for 5 min on an orbital shaker. The supernatant containing the intestinal crypts was centrifuged at 800*g* for 5 min at 4 °C. After being washed in ADMEM+++ and centrifuged, the pellet was resuspended in Matrigel (Corning, 354230) and plated in presence of Primocin (Invivogen, NC9141851) and ROCKi. The recipe for the medium is in Supplementary Table 3.

#### Human fetal tissue-derived kidney tubule organoids

The process was adapted following a previously published protocol for deriving adult tubuloids^14^. Briefly, fetal kidneys were collected, washed in ice-cold HBSS and minced to isolate the cortical tissue. The tissue was washed in 10 ml basal medium and the supernatant was removed when the tissue pieces settled at the bottom of the tube. After being washed several times in ADMEM+++, the tubule fragments were isolated by 1 mg ml^−1^ collagenase digestion (C9407, Sigma) on an orbital shaker for 30–45 min at 37 °C. Fragments were further washed in basal medium with 2% FBS and centrifuged at 300*g* for 5 min at 4 °C. Pellets were resuspended in Matrigel (Corning, 354230) and cultured in kidney organoid medium (Supplementary Table 3) supplemented with ROCKi and Primocin (Invivogen, NC9141851).

#### Human fetal tissue-derived lung organoids

Fetal lung tissue was processed by adapting a previously published protocol^11^. Briefly, fetal lungs were minced and washed in ADMEM+++. Tissue fragments were digested in ADMEM+++ containing 1 mg ml^−1^ collagenase (C9407, Sigma) on an orbital shaker at 37 °C for 30–60 min. The digested tissue was shaken vigorously and strained over a 100-μm filter. Tissue fragments were washed in ice-cold basal medium with 2% FBS and centrifuged at 300*g* for 5 min at 4 °C. Supernatant was discarded and pellet was resuspended in Matrigel (Corning, 354230) and cultured in lung medium (Supplementary Table 3) supplemented with ROCKi and Primocin (Invivogen, NC9141851).

#### Human fetal tissue-derived stomach organoids

Fetal stomach organoids were isolated from specimens following an established dissociation protocol^15^. Briefly, stomachs were cut open and mucus was removed with a glass coverslip and mucosa was stripped from muscle layer. Mucosa samples were cut into pieces of 3–5 mm and washed in HBSS until the supernatant was clear. The tissue was incubated in chelating buffer supplemented with 2 mM EDTA for 30 min at room temperature. Tissue fragments were squeezed with a glass slide to isolate the gastric glands, which were transferred in ADMEM+++, strained through at 40 μm and centrifuged at 300*g* for 5 min at 4 °C. The pellet was resuspended in Matrigel (Corning, 354230) and plated. Gastric medium (Supplementary Table 3) was added with ROCKi and Primocin (Invivogen, NC9141851).

#### Human tissue-derived placental and fetal bladder organoids

Fetal bladder or placental biopsies were isolated and washed with ice-cold HBSS. Briefly, the samples were minced and washed in ADMEM+++. Tissue fragments were digested in ADMEM+++ containing 1 mg ml^−1^ collagenase (C9407, Sigma), 2.4 U ml^−1^ dispase (Thermo Fisher, 17105041) and 0.1 mg ml^−1^ DNase (Merck, 260913) on an orbital shaker at 37 °C for 20–60 min. The digested tissue was then strained through at 70 μm followed by 40 μm. Tissue fragments were washed in ice-cold basal medium with 10% FBS in 50-ml Falcon tubes and centrifuged at 300*g* for 10 min at 4 °C. The supernatant was discarded and the pellet was resuspended in Matrigel (Corning, 354230) and cultured in expansion medium (Supplementary Table 3) supplemented with ROCKi and Primocin (Invivogen, NC9141851).

### Organoid cryopreservation and thawing

After 7–10 d of culture, organoids were dissociated enzymatically as described above. The final cell pellet was resuspended in 1:1 ADMEM+++ and freezing medium (80% FBS and 20% dimethylsulfoxide). Cryovials were stored at −80 °C overnight and then transferred to LN2 for long-term storage. For organoid thawing, cryovials were equilibrated on dry ice and then placed at 37 °C. Vial content was rapidly transferred to 15-ml Falcon tubes containing 9 ml ice-cold ADMEM+++, then centrifuged at 300*g* for 5 min at 4 °C. The supernatant was discarded and the pellet was resuspended in cold Matrigel (Corning, 354230). After 20 min of incubation at 37 °C, the medium was supplemented with ROCKi and replaced after 3 d.

### Evaluation of organoid formation efficiency and area

Organoid formation efficiency was determined by counting the number of organoids at P0. The total number of formed organoids per well was manually counted approximately 14 d after seeding of the AF cells in Matrigel. The efficiency was determined by calculating the total number of grown organoids divided by the number of viable single cells initially plated. The organoid area was determined by measuring the perimeter of each organoid in different ×5 fields acquired at the Zeiss Axio Observer A1 and using ImageJ software. The number of formed organoids over passages was calculated by counting the organoids in each ×5 field and normalized by field size.

### Organoid maturation/differentiation

For SiAFOs, after manual passaging, organoids were seeded in triplicate in Matrigel (Corning, 354230) and cultured in AFO expansion medium. After approximately 7 d, human small intestine medium was used (Supplementary Table 3) for 14 d. Basal culture medium was the same but without the addition of CHIR99021. DAPT (Notch inhibitor) 10 μm was added to basal culture medium for 48 h to stimulate differentiation. For KAFOs, after either manual or enzymatic passaging, organoids were seeded in triplicate in Matrigel and cultured in AFO expansion medium. After approximately 7–10 d, distal/collecting duct kidney differentiation medium (Supplementary Table 3) was used for 14 d^62^. For LAFOs, after manual passaging, organoids were seeded in triplicate in Matrigel and cultured in AFO expansion medium for approximately 10 d. For lung proximal differentiation, PneumaCult ALI Medium (Stem Cell Technologies, 05001) was used for 14 d. For distalization, organoids were exposed for 14 d to a previously reported medium^63^ (Supplementary Table 3).

### Intestinal ring formation in collagen hydrogel

The assay was adapted from a previously published protocol^64^. SiAFOs were expanded for 7 d in AFO expansion medium. After the medium was removed and the wells were washed once with PBS, Matrigel droplets were transferred to 1% BSA pre-coated Eppendorf tubes and dissolved in Cell Recovery Solution (Corning, 354253) for 45 min on ice. Organoids were centrifuged at 300*g* for 5 min at 4 °C and the supernatant was removed. The organoids were collected and resuspended in 120 μl collagen hydrogel (collagen type I 0.75 mg ml^−1^, DMEM-F12 1×, HEPES 1 M, MilliQ to volume, pH 7) and plated in an ultra-low adherent 24-well plate making a circular shape around the edges of the well. The plate was incubated for 30 min at 37 °C and medium was added in the middle of the well to allow homogeneous detachment of the collagen ring. Intestinal rings were cultured in suspension in intestinal medium for 10 d.

### Whole-mount immunofluorescence

Before fixation, organoids were retrieved from Matrigel using Cell Recovery Solution for 45 min on ice. Organoids were collected in a 15-ml tube precoated with 1% BSA in PBS and fixed with 4% PFA for 20 min at room temperature. Samples were washed three times with PBS for 5 min and spun down at 300*g* for 5 min at 4 °C. Whole-mount immunostaining was performed by blocking and permeabilizing the organoids with PBS-Triton X-100 0.5% with 1% BSA for 1 h at room temperature. Primary antibodies were incubated in blocking/permeabilization buffer for 24 h at 4 °C in agitation. After being extensively washed with PBS-Triton 0.2%, organoids were incubated with secondary antibodies and Hoechst overnight at 4 °C in agitation. After incubation, organoids were further washed and resuspended in PBS in preparation for confocal imaging. For tissue clearing of the SiAFO ring, a previously published protocol was adapted^65^. EdU staining was performed with the Click-iT EdU Alexa Fluor 568 Imaging kit (Life Technologies) following the manufacturer’s protocol. A full list of antibodies is available in Supplementary Table 4.

### Image acquisition

Phase-contrast images were acquired using Zeiss Axio Observer A1 and Zeiss ZEN (v.3.1) software. IF images of whole-mount staining and sections were acquired on a Leica SP5 or a Zeiss LSM 710 confocal microscope using ×20, ×25, ×40 and ×63 immersion objectives. Image analysis and z-stack projections were generated using ImageJ (https://imagej.nih.gov/ij/). Videos of SiAFO rings were processed using Imaris (v.2).

### X-ray PC-CT

The imaging of the organoids was performed using PC-CT at beamline I13-1 (coherence branch) of the Diamond Light Source (www.diamond.ac.uk). The X-ray energy was 9.7 keV and the system resolution was 1.6 μm. The organoids were imaged embedded in HistoGel (Epredia HistoGel). The PC-CT scan entailed the acquisition of 2,000 equally spaced projections through a 180º rotation of the specimen. The total scan time was approximately 1 h. The ‘single image’ phase retrieval operation^66^ was applied to the acquired projections, with the estimated phase to attenuation ratio (referred to as δ:β ratio) set at 250. Both phase retrieval and tomographic slice reconstructions were performed using Savu^67^ and 3D images were generated using Drishti^68,69^.

### microCT

After 10 d of culture, SiAFO rings were collected and processed for microCT scanning. Rings were fixed for 1 h in 4% PFA and extensively washed in PBS. The specimen was iodinated overnight by immersion in 1.25% potassium triiodide in 10% formalin solution. After being rinsed in deionized water, the sample was wrapped in laboratory wrapping film and mounted in HistoGel (Epredia, HG-4000-012) in a 1.5-ml tube. MicroCT scanning was performed using a Nikon Med-X microCT scanner (Nikon Metrology). The specimen was mounted and held in pace using a drill chuck to ensure centralized rotational positioning. Whole specimen scans were acquired using an X-ray energy of 120 kV, current of 50 μA, exposure time of 1,000 ms, four frames per projection, a detector gain of 24 dB and an optimized number of projections of 2,258. A tungsten target was used and an isotropic voxel size of 3.57 μm was achieved. Reconstructions were carried out using modified Feldkamp filtered back projection algorithms with CTPro3D (Nikon, Metrology, v.XT 5.1.43) and post-processed using VGStudio MAX (Volume Graphics, v.3.4).

### SiAFO dipeptidyl peptidase IV and disaccharidase assays

For both assays organoids were plated in 48-well plates, 15 μl basement membrane extract per well, in triplicate. For the dipeptidyl protease assay, organoids were washed in PBS and then incubated at 37 °C with 200 μl per well in Gly-Pro p-nitroanilide hydrochloride (Sigma, G0513) dissolved in PBS at a concentration of 1.5 mM (or PBS alone in control wells). During incubation, samples were agitated on an orbital shaker (60 r.p.m.) and supernatants were sampled at 20, 40 and 60 min. Absorbance (415 nm) was measured with a plate reader (Bio-Rad) and the concentration was determined by comparison to a 4-nitroaniline (Sigma, 185310) standard curve (0–200 µg ml^−1^) and normalized per mg organoid lysate protein (Pierce BCA Protein Assay kit, Thermo Scientific). For the disaccharidase assay, basement membrane extract was removed by adding Cell Recovery Solution for 40 min at 4 °C and organoids were placed in 1.5-ml Eppendorf tubes, one well per Eppendorf. Organoids were washed once in PBS and then incubated at 37 °C with 200 µl per Eppendorf of 56 mM sucrose in PBS (or PBS alone for controls). During incubation, samples were agitated on an orbital shaker (60 r.p.m.). Supernatants were collected at 0, 30, 60, 90 and 120 min and sampled for glucose detection using the Amplex Red glucose/glucose oxidase assay kit (Thermo Fisher, A22189) according to the manufacturer’s protocol. Briefly, 50 µl of the reaction working solution was added to 50 µl of the test samples (diluted 1:4 with 1× reaction buffer) in a 96-well black flat-bottom microtiter plate in duplicate and incubated in the dark for 30 min at room temperature. Fluorescence (excitation 535 nm and emission 590 nm) was measured using a Tecan microplate reader (Infinite M1000 PRO). Glucose concentration was determined by comparison to a glucose standard curve and normalized per mg organoid lysate protein (Pierce BCA Protein Assay kit, Thermo Scientific, 23225).

### KAFO potassium ion channel assay

KAFOs were expanded at least in triplicate in 96-well plates (10 µl Matrigel per well). FluxOR II Green Potassium Ion Channel Assay was performed according to the manufacturers’ instructions (F20017, Thermo). Briefly, medium was removed and 80 µl 1× loading buffer was added to each well and incubated for 30 min at room temperature and 30 min at 37 ºC to facilitate dye entry. After removing the loading buffer, 80 µl assay buffer was added to each well. A microplate reader (SoftMax Pro v.7.1.2) was set at an excitation wavelength of 480 nm and an emission wavelength of 545 nm and recorded every 5 s for 5 min. After 5 min of plate recording, voltage-gated channels were stimulated with 20 µl High Potassium Stimulus Buffer containing 2 mM thallium sulfate (Tl_2_SO_4_) and 10 mM potassium sulfate (K_2_SO_4_). The plate was read once again every 5 s for 5 min. The average of replicates was normalized to the control (assay buffer) and the number of cells.

### KAFO epithelial barrier integrity assay

Expanded KAFOs were collected in Cell Recovery Solution for 45 min on ice in 15-ml tubes precoated with 1% BSA. Afterwards, organoids were equally divided into two tubes for the two conditions (EDTA^+^ and EDTA^−^), washed once with ADMEM+++ and centrifuged at 300*g* for 5 min at 4 °C. The supernatant was removed and, to disrupt the epithelial barrier integrity, organoids were incubated with or without 200 µl 4 mM EDTA in PBS on ice for 15 min. Organoids were centrifuged at 60*g* for 5 min and the EDTA solution was removed. Subsequently, 200 µl 500 µg ml^−1^ 2–5 kDa inulin-FITC^70^ (Sigma, F3272) resuspended in ADMEM+++ was added to both conditions. Organoids were then incubated for 60 min at 37 °C and directly imaged using an LSM 710 Zeiss confocal microscope. The proportion of organoids with intact barrier integrity (organoids without inulin signals inside the lumen) of each group was calculated for quantification analysis.

### Ciliary beat frequency analysis

Organoids were seeded into eight-well glass-bottom slides (ibidi, 80806) and differentiated toward the lung proximal lineage for 14 d as described above. For CBF analysis, motile cilia grown inside organoids were observed using an inverted microscope system (Nikon Ti-U; Nikon NIS-Elements v.5.41.02 software) with a digital high-speed video camera (Prime BSI Express, Teledyne Photometrics). Videos were recorded at a rate of approximately 178 frames per second using a ×20 objective with ×1.5 magnifier. For each subject a minimum of five organoids were studied. The video of each organoid was divided into 16 small regions of interest (256 × 256) for analysis. The time taken for five full ciliary beats was recorded. Clearly visible cilia from every small region of interest were counted for five full beats and the number of high-speed video frames for five full beats was noted. CBF (Hz) was calculated as (178/(number of frames for five beats)) × 5.

### TEM

Organoids in the matrix were fixed in a mix of 2.5% glutaraldehyde and 4% paraformaldehyde in 0.1 M Sorensen’s buffer, pH 7.3, and washed with 0.1 M Sorensen’s buffer. They were postfixed in 1% aqueous solution of osmium tetroxide, washed and dehydrated through an increasing series of ethanol solutions, followed by propylene oxide (Merck). Organoids were embedded in TAAB812 resin (TAAB Laboratory Equipment) and cut to approximately 70-nm thick sections using a Leica UC7 Ultramicrotome (Leica Microsystems). Sections were collected onto copper mesh grids and contrasted for 2 min with 4% uranyl acetate solution in methanol (VWR), followed by 2 min in lead citrate (Reynolds’ solution). Samples were viewed on a JEOL JEM-1400 TEM (JEOL) with an accelerating voltage of 120 kV. Digital images were collected with a Xarosa digital camera using Radius software (both from EMSIS).

### Flow cytometry

Cold-stored AF was processed as described above for viable cell sorting. Viable cells were resuspended in FACS blocking buffer (FBB) and incubated for 30 min at 4 °C with the following fluorochrome-conjugated antibodies: APC/Fire 750 anti-human CD324 (E-cadherin) (BioLegend, 324122, 5 µl per tube), APC/Fire 750 anti-human CD326 (EpCAM) (BioLegend, 324233, 5 µl per tube). Cells were washed in 10 ml FBB and analyzed using a BD FACSymphony A5 (BD FACSDiva v.8.0.1 software). Data were processed on FlowJo (v.10.15).

### RNA isolation and RT–qPCR

Organoids were collected from Matrigel with Cell Recovery Solution for 45 min on ice. Cells were then washed in ice-cold PBS to remove leftover Matrigel. Organoids were centrifuged at 300*g* for 5 min at 4 °C and the supernatant was discarded. Pellet was resuspended and lysed with RLT buffer (QIAGEN). Total RNA was isolated with an RNeasy Micro or Mini kit (QIAGEN) following the manufacturer’s instructions. RNA concentration was quantified using a NanoDrop (Thermo). Complementary DNA was prepared using High-Capacity cDNA Reverse Transcription kit (Applied Biosystems, 4368813). Quantitative real-time PCR detection was performed using PowerUp SYBR Green Master Mix (Applied Biosystems, A25742) and StepOnePlus Real-Time PCR System (Applied Biosystems). Assays for each sample were run in triplicate and were normalized to the housekeeping gene β-actin. Primer sequences are listed in Supplementary Table 5.

### Bulk RNA sequencing

RNA was extracted as described above and stored at −80 °C until processing. NEBNext Low-Input RNA library preparation and sequencing were performed by the UCL genomics facility. Single-end bulk RNA sequencing was conducted on an Illumina NextSeq 2000. Then, 100 cycles were run to achieve an average of 5 million reads per sample.

### Transcriptome bioinformatics analysis

Quality control was conducted on FASTQ raw sequences using v.0.11.9 FastQC (https://github.com/s-andrews/FastQC). Then, TrimGalore! v.0.6.6 (https://github.com/FelixKrueger/TrimGalore) was used to trim low-quality reads (quality 20 and length 70). STAR v.2.7.1a (https://github.com/alexdobin/STAR) was applied to align FASTQ sequences to the National Center for Biotechnology Information (NCBI) human reference genome GRCh38.p13 (www.ncbi.nlm.nih.gov/assembly/GCF_000001405.39/). featureCounts v.1.6.3 (10.1093/bioinformatics/btt656) quantified the expression of individual genes to generate the raw count matrix, using the GRCh38.104 gene annotation (www.ensembl.org/Homo_sapiens/Info/Index). Default parameters were used for both alignment and quantification. The generated count matrix was further processed with a custom R script. Genes with fewer than ten reads across three samples were removed. Gene IDs were included using the added BioMart package (www.ensembl.org/info/data/biomart/biomart_r_package.html). CPM normalization was completed with the edgeR package (www.bioconductor.org/packages/release/bioc/html/edgeR.html).

ComBat_seq batch correction (rdrr.io/bioc/sva/man/ComBat_seq.html) was applied between the five batches. ggplot2 was used for graph generation, including PCA and dot plots generated from the normalized CPM matrix. Clustered heat maps were generated with pheatmap (cran.r-project.org/web/packages/pheatmap/) to enable hierarchical comparisons.

For the comparison analyses, the pheatmap hierarchical clustering DESeq2 (bioconductor.org/packages/release/bioc/html/DESeq2.html) with standard parameters was used to determine DEGs. A volcano plot was generated with ggplot2 to highlight statistically significant DEGs. Metascape Gene Annotation and Analysis Resource v.3.5 (metascape.org) was used to determine Gene Ontology pathway activation using the DEGs identified previously. A log fold change cutoff of 1 or 2 was applied as stated. An adjusted *P* value of 0.05 was used as the significance threshold.

### scRNA-seq of AF cells

Viable AF cells were isolated using FACS as described above. To preserve AF cell heterogeneity, we did not gate by forward or side scatter. Hoechst was used to identify nucleated cells and exclude debris; PI was used to identify dead and damaged cells. Viable cells were then immediately processed for cDNA library preparation. Library generation was conducted following the 10x Genomics Chromium Next GEM Single Cell 3ʹ Reagent kits v.3.1 (Dual Index). Libraries were sequenced using NovaSeq 6000. Data processing was conducted with v.6.0.1 CellRanger. Ambient RNA correction was carried out with CellBender v.0.2.2 on standard parameters (Broad Institute; github.com/broadinstitute/CellBender). Analysis was performed with Seurat v.4.1.1 within a custom R script that was used for further downstream processing. Cells with fewer than 150 features were removed to prevent doublets or cells of low quality. There were 38,880 total cells and 33,934 cells after filtering. A single small cluster with high mitochondrial percentage genes was removed. No cell cycle correction was carried out. Normalization was then carried out using the NormalizeData function, with a logNormalize method and a scale factor of 10,000. Batch correction was completed through Seurat’s IntegrateData function after assessing for integration anchors based on the 2,000 most variable features. The object was scaled using ScaleData, RunPCA and FindNeighbors determined for 20 principal components. UMAPs and violin plots were generated using ggplot2 and normalized gene expression was always shown, with violin plots showing averaged normalized gene expression within the identified epithelial cluster. SingleR^34^ v.1.6.1 was used to label the epithelial cluster. A single-cell experiment (github.com/drisso/SingleCellExperiment) object was analyzed using Human Primary Cell Atlas Data (www.humancellatlas.org), accessed via celldex (github.com/LTLA/celldex). Determining the tissue of origin for the AFEpCs was conducted through scGSEA, using escape package v.1.12.0 (bioconductor.org/packages/escape) with the enrichIt function on standard parameters and the C8 cell type signature gene set from the Broad Institute as a reference (gsea-msigdb.org/gsea/msigdb). The Seurat function ModuleScore was then used to score these tissue cells for a range of progenitor markers (‘Results’).

### Single-cell RNA sequencing of organoids

Organoids were expanded or differentiated according to the above protocols. After washing with PBS, organoids were collected in Cell Recovery Solution for 45 min at 4 ºC for Matrigel removal and disaggregated to single cells by incubation with TrypLE for 7–10 min at 37 ºC. Cells were washed with FBB containing 1% FBS and 0.5 mM EDTA in PBS and centrifuged at 300*g* for 5 min at 4 ºC. Cells were resuspended in FBB and cell viability was confirmed using a Live/Dead Acridine Orange/PI fluorescent Luna cell counter. Cells were prepared at 1,000 cells per µl and cDNA library generation was completed following the 10x Genomics Chromium Next GEM Single Cell 3ʹ kit v.3.1 (Dual Index). Sequencing was completed by the UCL genomics facility. Data were pre-processed using v.6.0.1 CellRanger scRNA-seq. Upon formation of the count matrices, analysis was continued within a custom R script using Seurat v.4.1.1. A total of 41 organoids were sequenced across six lanes, meaning that deconvolution was required. No batch correction or cell-cycle correction was carried out. Deconvolution of the data was achieved in a two-step process. Initially, single-nucleotide polymorphism profiles were generated using CellSNP-lite v.1.2.0 from the same AFO bulk RNA-seq data for organoids from the same samples as sequenced through scRNA-seq. The NCBI Human single-nucleotide polymorphism dataset was used as a reference (https://ftp.ncbi.nlm.nih.gov/snp/organisms/human_9606/VCF/00-common_all.vcf.gz). The scRNA-seq data were then deconvoluted using Vireo v.0.5.6 on a cell-by-cell basis. The total number of cells was 36,463. All cells not confidently assigned (as determined by Vireo) were excluded (*n* = 3,805, 9.8%). All barcodes identified as doublets by Vireo were removed (*n* = 5,356, 13.8%). Differentiated/mature organoids were sequenced in different lanes from the undifferentiated to enable separation. Cell barcode patient separations are provided as supplementary data on the Gene Expression Omnibus. A 30% mitochondrial gene cutoff was applied to the SiAFOs but this was deemed unneeded for KAFOs or LAFOs. SingleR v.1.61 was used to label epithelial cells as discussed previously. Labeling of the different epithelial cell types present was conducted differently for each tissue. For the SiAFO, CellTypist (www.celltypist.org) was used with Cells_Intestinal_Tract as input^71^. This was performed in conjunction with manual marker analysis to label cluster by cluster. For LAFOs, CellTypist was also used with three input references: Cells_Fetal_Lung^41^, Cells_Lung_Airway^72^ and Human_Lung_Atlas^73^, in conjunction with marker analysis. KAFOs were labeled with the automatic kidney-labeling tool DevKidCC (github.com/KidneyRegeneration/DevKidCC)^44^, combined with marker analysis.

### Statistics and reproducibility

Statistical analysis was conducted on data from at least three independent experimental or biological replicates wherever possible, as stated in the figure legends. Results are expressed as mean ± s.d. or s.e.m., as the median and quartiles (25% and 75% percentiles) or 95% CI range. Statistical significance was analyzed using unpaired or paired *t*-tests for comparisons between two different experimental groups. Statistical significance was assessed using one-way or two-way ANOVA with Dunnett’s, Holm–Šídák or Tukey’s multiple-comparisons test for analysis among more than two groups. *P < 0.03, **P < 0.002, ***P < 0.0002, ****P < 0.0001 were considered significant. Exact *P* values are stated in each figure legend where appropriate. Statistical analysis was carried out using R and GraphPad Prism v.10 software.

### Reporting summary

Further information on research design is available in the Nature Portfolio Reporting Summary linked to this article.

## Online content

Any methods, additional references, Nature Portfolio reporting summaries, source data, extended data, supplementary information, acknowledgements, peer review information; details of author contributions and competing interests; and statements of data and code availability are available at 10.1038/s41591-024-02807-z.

## Supplementary information

Supplementary Information Supplementary Tables 1–5.

Reporting Summary

Supplementary Video 1 The 3D reconstruction of microCT imaging performed on a whole SiAFO ring.

Supplementary Video 2 Whole-mount 3D immunofluorescent staining and tissue clearing of SiAFO ring (nuclei in blue, KRT20 in red, ITGβ4 in green and CHGA in yellow).

Supplementary Video 3 High-speed video microscopy of beating cilia and whirling mucus and debris within the lumen of proximally differentiated LAFOs.

Supplementary Video 4 Phase-contrast video of beating cilia and whirling mucus and debris within the lumen of proximal CDH LAFOs.

## Source data

Source Data Fig. 1 Flow cytometry data analysis.

Source Data Fig. 2 Statistical source data.

Source Data Fig. 3 Statistical source data.

Source Data Fig. 4 Statistical source data.

Source Data Fig. 5 Statistical source data.

Source Data Fig. 6 Statistical source data.

Source Data Extended Data Fig. 1 FACS data analysis.

Source Data Extended Data Fig. 2 Statistical source data.

Source Data Extended Data Fig. 3 Statistical source data.

Source Data Extended Data Fig. 5 Statistical source data.

Source Data Extended Data Fig. 6 Statistical source data.

## Data Availability

Raw individual-level data and combined processed data of the bulk RNA sequencing (AFOs, TFOs and fetal tissue-derived organoids) and scRNA-seq (AF and AFOs) have been uploaded to the NCBI Gene Expression Omnibus (GSE220994). These data are openly available with no restriction or time limit. Questions or additional requests can be directed to the corresponding authors. Source data are provided with this paper.

## Code availability

Code is available on Zenodo at 10.5281/zenodo.8124205. This includes R scripts used for annotation, downstream analysis and visualization. This code is openly available with no restriction or time limit. Direct questions and additional requests can be directed to the corresponding authors.
