# Author Correction: Single-cell guided prenatal derivation of primary fetal epithelial organoids from human amniotic and tracheal fluids

**DOI:** 10.1038/s41591-025-03504-1

**Published:** 2025-01-21

**Authors:** Mattia Francesco Maria Gerli, Giuseppe Calà, Max Arran Beesley, Beatrice Sina, Lucinda Tullie, Kylin Yunyan Sun, Francesco Panariello, Federica Michielin, Joseph R. Davidson, Francesca Maria Russo, Brendan C. Jones, Dani Do Hyang Lee, Savvas Savvidis, Theodoros Xenakis, Ian C. Simcock, Anna A. Straatman-Iwanowska, Robert A. Hirst, Anna L. David, Christopher O’Callaghan, Alessandro Olivo, Simon Eaton, Stavros P. Loukogeorgakis, Davide Cacchiarelli, Jan Deprest, Vivian S. W. Li, Giovanni Giuseppe Giobbe, Paolo De Coppi

**Affiliations:** 1 Department of Surgical Biotechnology, Division of Surgery and Interventional Science, University College London, London, UK; 2 Great Ormond Street Institute of Child Health, University College London, London, UK; 3 Politecnico di Milano, Milan, Italy; 4 Stem Cell and Cancer Biology Laboratory, The Francis Crick Institute, London, UK; 5 Armenise/Harvard Laboratory of Integrative Genomics, Telethon Institute of Genetics and Medicine, Pozzuoli, Italy; 6 Elizabeth Garrett Anderson Institute for Women’s Health, University College London, London, UK; 7 Department of Development and Regeneration, Woman and Child and UZ Leuven Clinical Department of Obstetrics and Gynaecology, KU Leuven, Leuven, Belgium; 8 Department of Medical Physics and Biomedical Engineering, University College London, London, UK; 9 Department of Radiology, Great Ormond Street Hospital, London, UK; 10 Department of Respiratory Sciences, University of Leicester, Leicester, UK; 11 Specialist Neonatal and Paediatric Surgery, Great Ormond Street Hospital for Children NHS Foundation Trust, London, UK; 12 Department of Translational Medicine, University of Naples Federico II, Naples, Italy; 13 Genomics and Experimental Medicine Program, Scuola Superiore Meridionale, Naples, Italy; 14 Medical and Surgical Department of the Fetus, Newborn and Infant, Ospedale Pediatrico Bambino Gesù, IRCCS, Rome, Italy; 15 NIHR Great Ormond Street Hospital Biomedical Research Centre, London, UK

**Keywords:** Stem cells, Regenerative medicine, Stem-cell research, Experimental models of disease

Correction to: *Nature Medicine* 10.1038/s41591-024-02807-z, published online 4 March 2024.

In the version of this article initially published, there were inconsistences, where in the Methods “AF collection and isolation of the viable cell fraction” paragraph, the first sentence, now reading “AF samples (amniocenteses and amniodrainages) were collected from UCLH FMU and UZ Leuven as part of standard patient clinical care”, was initially preceded by “Euploid,” though the sample contained two chromosomal abnormalities; and in Supplementary Table 1, where for sample code HO680, the sex was listed as female following prenatal clinical data, whereas the sample was identified and processed in further analysis as male. The changes have been made in Supplementary Table 1 and in the HTML and PDF versions of the article.

## Supplementary information

Supplementary Tables

