# Supplementary material for: Single-cell guided prenatal derivation of primary fetal epithelial organoids from human amniotic and tracheal fluids
Source: Nat Med. 2024 Mar 4;30(3):875–87. doi: 10.1038/s41591-024-02807-z (PMC10957479; doi:10.1038/s41591-024-02807-z)
Supplement: Supplementary file 1 — Supplementary Tables 1–5. [file 41591_2024_2807_MOESM1_ESM.pdf]

nature medicine

Article

https://doi.org/10.1038/s41591-024-02807-z

Single-cell guided prenatal derivation of primary fetal epithelial organoids from human amniotic and tracheal fluids

In the format provided by the authors and unedited

Supplementary Table 1: List of amniotic and tracheal fluid samples.

AF = Amniotic Fluid, TF = Tracheal Fluid, CDH = Congenital Diaphragmatic Hernia, TTTS = Twin to Twin Transfusion Syndrome, FETO = Fetoscopic Endoluminal Tracheal Occlusion , M = Male, F = Female.

Sample code	GA	Type of fluid	Procedure	Reason for procedure	Associated morbidity	Sex	AFO generated	LAFO generated	KAFO generated	SiAFO generated	AFO RNAseq	AFO scRNAseq	AF scRNAseq
15993	16+0	AF	TOP	HDBR fetus	/	M	Y	N	N	Y	Y	Y	N
#10 CDH	31+4	AF	Pre-FETO	FETO	CDH	F	N	N	N	N	N	N	N
#10 CDH	31+4	TF	Pre-FETO	FETO	CDH	F	Y	Y	N	N	Y	Y	N
#10 SB	23+6	AF	Fetal surgery	/	Spina bifida	M	Y	Y	Y	N	Y	N	N
#11 CDH	27+6	AF	Pre-FETO	FETO	CDH-L	M	Y	Y	N	N	Y	N	N
#11 CDH	27+6	TF	Pre-FETO	FETO	CDH-L	M	N	N	N	N	N	N	N
#11 SB	24+1	AF	Fetal surgery	/	Spina bifida	F	Y	/	/	/	N	N	N
#14 CDH	27+2	AF	Pre-FETO	FETO	CDH-L	F	Y	N	Y	N	Y	N	N
#14 CDH	27+2	TF	Pre-FETO	FETO	CDH-L	F	N	N	N	N	N	N	N
#15 CDH	27+6	AF	Pre-FETO	FETO	CDH-L	M	N	N	N	N	N	N	N
#15 CDH	27+6	TF	Pre-FETO	FETO	CDH-L	M	N	N	N	N	N	N	N
#16 CDH	28+5	AF	Pre-FETO	FETO	CDH-L	M	Y	/	/	/	N	N	N
#16 CDH	28+5	TF	Pre-FETO	FETO	CDH-L	M	N	N	N	N	N	N	N
#17 CDH	29	AF	Pre-FETO	FETO	CDH-L	M	Y	Y	N	N	Y	N	N
#17 CDH	29	TF	Pre-FETO	FETO	CDH-L	M	N	N	N	N	Y	N	N
#18 CDH	29+5	AF	Pre-FETO	FETO	CDH-L; IUGR	M	Y	/	/	/	N	N	N
#19 CDH	27+6	AF	Pre-FETO	FETO	CDH-L	F	Y	/	/	/	N	N	N
#19 CDH	27+6	TF	Pre-FETO	FETO	CDH-L	F	N	N	N	N	N	N	N
#2 CDH	29+3	AF	Pre-FETO	FETO	CDH-R	F	Y	Y	N	N	Y	N	N
#2 CDH	29+3	TF	Pre-FETO	FETO	CDH-R	F	N	N	N	N	N	N	N
#20 CDH	28+4	AF	Pre-FETO	FETO	CDH-L	M	Y	/	/	/	N	N	N
#20 CDH	28+4	TF	Pre-FETO	FETO	CDH-L	M	N	N	N	N	N	N	N
#4 CDH	28+6	AF	Pre-FETO	FETO	CDH-L	F	Y	N	N	N	N	N	N
#5 CDH	29+4	AF	Pre-FETO	FETO	CDH-R	M	Y	Y	Y	N	Y	N	N
#6 SB	25+0	AF	Fetal surgery	/	Spina Bifida	M	Y	Y	Y	N	Y	Y	N
#9 CDH	33+6	AF	Post-FETO	FETO	CDH-L	M	Y	Y	N	N	Y	N	N
#9 CDH	33+6	TF	Post-FETO	FETO	CDH-L	M	Y	Y	N	N	Y	N	N
#9 SB	25+6	AF	Fetal surgery	/	Spina bifida	F	Y	Y	Y	N	Y	N	N
HO680	19+0	AF	Amniodrainage	TTTS	None	F	/	/	/	/	/	/	Y
HO686	19+0	AF	Amniocentesis	/	/	/	Y	/	/	/	/	/	N
HO687	34+4	AF	Amniodrainage	/	Oesophageal atresia	M	Y	Y	Y	N	Y	N	Y
HO689	19+1	AF	Amniodrainage	/	None	M	N	/	/	/	/	/	Y
HO695	15+3	AF	Amniocentesis		Trisomy 18	M	/	/	/	/	/	/	Y
HO696	32+0	AF	/	/	Congenital Heart Disease	M	/	/	/	/	/	/	Y
HO713	16+0	AF	Amniodrainage	/	/	F	/	/	/	/	/	/	Y
HO719	19+2	AF	Amniodrainage	TTTS	One twin with abnormal brain	F	/	/	/	/	/	/	Y
HO721	18+0	AF	Amniodrainage	MCDA twins TTTS (Stage2)	Surviving twin: perinatal brain injury and limb anomaly	F	Y	N	Y	N	Y	N	N
HO724	24+0	AF	Amniodrainage	TTTS (Stage3)	Normal triplets	M	Y	N	Y	N	Y	Y	N
HO725	21+0	AF	Amniodrainage	TTTS	Both twins miscarried 6 days after procedure	M	/	/	/	/	/	/	Y
HO727	25+4	AF	Fetal surgery	/	Spina Bifida	F	Y	Y	Y	N	Y	Y	N
HO728	20+5	AF	Amniocentesis	/	Spina Bifida	F	Y	N	Y	N	Y	N	N
HO730	28+5	AF	Pre-FETO	FETO	CDH-R	M	Y	Y	N	N	Y	N	N
HO730	28+5	TF	Pre-FETO	FETO	CDH-R	M	Y	Y	N	N	Y	N	N
HO730	32+6	AF	Post-FETO	FETO	CDH-R	M	N	N	N	N	N	N	N
HO730	32+6	TF	Post-FETO	FETO	CDH-R	M	Y	Y	N	N	Y	Y	N
HO734	22+3	AF	Amniodrainage	TTTS (Stage1)	One normal surviving twin, one fetal loss	M	Y	N	Y	N	Y	N	N
HO741	30+6	AF	Amniodrainage	Late onset Hydrops	In utero death	F	Y	N	Y	N	Y	N	Y
HO742	22+2	AF	Amniodrainage	TTTS (Stage4)	One normal surviving twin, one fetal loss	M	Y	N	Y	N	Y	Y	N
HO744	27+2	AF	Amniodrainage	MCDA-Twins TTTS (Stage4)	One normal surviving twin, one fetal loss	M	Y	Y	Y	N	Y	Y	Y
HO754	20+6	AF	Amniodrainage	TTTS (Stage2)	One normal surviving twin, one fetal loss	F	Y	Y	Y	N	Y	N	N
HO758	30+2	AF	Amniodrainage	Polyhydramnios	Parital duplication Chr7; TOP	M	Y	Y	Y	N	Y	N	Y
HO760	28+1	AF	Pre-FETO	FETO	CDH-L	F	Y	Y	Y	N	Y	Y	N
HO760	28+1	TF	Pre-FETO	FETO	CDH-L	F	N	N	N	N	N	N	N
HO768	29+6	AF	Pre-FETO	FETO	CDH-L	F	Y	/	/	/	N	N	N
HO768	29+6	TF	Pre-FETO	FETO	CDH-L	F	N	N	N	N	N	N	N
HO768	33+6	TF	Post-FETO	FETO	CDH-L	F	Y	Y	N	N	Y	N	N
HO768	33+6	AF	Post-FETO	FETO	CDH-L	F	Y	Y	N	N	Y	Y	N
HO773	17+0	AF	Amniocentesis	TTTS (Stage2)	Normal twin	M	Y	Y	N	Y	Y	Y	N
HO774	22	AF	Amniodrainage	laser TTTS (Stage2)	/	M	N	/	/	/	/	/	Y
HO775	24+5	AF	Fetal surgery	/	Spina bifida	F	Y	N	Y	N	Y	N	N
HO777	16+3	AF	Amniocentesis	MCDA-Twins post-laser	/	F	Y	Y	N	N	Y	Y	N
HO778	23+5	AF	Amniodrainage	MCDA twins laser TTTS (Stage3)	None	M	Y	Y	Y	N	Y	N	N
HO789	20+3	AF	Amniodrainage	MCDA TTTS (Stage3)	Gross poly twin A	M	Y	Y	N	N	Y	N	N
HO794	23+6	AF	Amniodrainage	MCDA twins laser TTTS	/	F	Y	/	/	/	N	N	N
HO797	18+2	AF	Amniocentesis	/	Severe bilateral ventriculomegaly	F	N	/	/	/	/	/	N
HO821	19+1	AF	Amniodrainage	MCDA laser TTTS (Stage2)	/	/	Y	/	/	/	N	N	N
HO822	19+3	AF	Amniodrainage	MCDA laser TTTS (Stage1)	/	/	Y	/	/	/	N	N	N
HO823a	29+2	AF	Pre-FETO	FETO	CDH	M	Y	Y	/	/	N	N	N
HO823a	29+2	TF	Pre-FETO	FETO	CDH	M	Y	/	/	/	N	N	N
HO823b	34+1	AF	Post-FETO	FETO	CDH	M	N	N	N	N	N	N	N
HO823b	34+1	TF	Post-FETO	FETO	CDH	M	Y	Y	/	/	N	N	N
HO827	28+3	AF	Amniodrainage	Polyhydramnios and moderate bilateral pleural effusion	generalised skin oedema	M	Y	/	/	/	N	N	N

Supplementary Table 2: List of sample size.
AF = Amniotic Fluid, TF = Tracheal Fluid, SiAFO = Small intestinal AF organoids, KAFO = Kidney AF organoids, LAFO = Lung AF organoids, CDH = Congenital Diaphragmatic Hernia.

**AF cells processed by scRNAseq**	33,934 cells from 12 AF samples
**Total AFO generated**	423 from 42 AF samples
**Total TFO generated**	137 from 7 TF samples
**Organoids sequenced by bulk RNAseq**	239 lines from 32 AF and 4 TF samples
**AFO**	121 lines from 23 AF samples
SiAFO	23 lines from 2 AF samples
KAFO	54 lines from 19 AF samples
LAFO (non-CDH)	43 lines from 12 AF samples
**CDH lung organoids**	53 lines from 8 AF and 4 TF samples
CDH LAFO	30 lines from 8 AF samples
CDH LTFO	23 lines from 4 TF samples
**Mature / differentiated organoids**	
Mature SiAFO	6 lines from 2 AF samples
SiAFO rings	5 rings from 2 AF samples
Differentiated KAFO	7 lines from 6 AF samples
Differentiated LAFO (non-CDH)	7 lines proximal; 7 lines distal from 7 AF samples
Differentiated CDH LAFO	3 lines proximal; 3 lines distal from 2 AF samples
Differentiated CDH LTFO	3 lines proximal; 3 lines distal from 2 TF samples
**Fetal tissue-derived organoids CT**	20 lines
**Organoids sequenced by scRNAseq**	
SiAFO	1,576 cells (3 lines from 2 AF samples)
Mature SiAFO	1666 cells (3 lines from 2 AF samples)
KAFO	1,467 cells (6 lines from 5 AF samples)
Differentiated KAFO	3,559 cells (3 lines from 3 AF samples)
LAFO (non-CDH)	1,966 cells (4 lines from 4 AF samples)
Differentiated LAFO (non-CDH)	3,371 cells proximal; 1,351 cells distal (3 lines from 3 AF samples
CDH LAFO	923 cells (3 lines from 2 AF samples)
CDH LTFO	954 cells (3 lines from 2 TF samples)
Differentiated CDH LAFO	1,478 cells proximal; 1,924 cells distal (3 lines from 2 AF samples)
Differentiated CDH LTFO	2,365 cells proximal; 4,166 cells distal (3 lines from 2 TF samples)

Supplementary Table 3: media composition

**AFO expansion medium**	
**Component**	**Final Concentration**
Advanced DMEM (Thermo 12634)	To volume
HEPES (Thermo 15630080)	10 mM
Glutamax (Thermo 35050061)	2 mM (1X)
Pen/Strep (Thermo 15140122)	1%
B-27 supplement minus Vitamin A (Thermo 12587010)	1X
n-acetylcysteine (Sigma A9165)	1.25 mM
Wnt-3A (Peprotech 315-20)	100 ng/mL
R-spondin 1 (Peprotech 120-38)	500 ng/mL
Noggin (Peprotech 120-10C)	100 ng/mL
EGF (Thermo PMG8043)	50 ng/mL
TGFb inhibitor (A83-01) (Sigma SML0788)	5 µM
GSK-3 inhibitor (CHIR 99021) (Tocris 4423)	3 µM
ROCK inhibitor Y-27632 (Tocris 1254) (Add to single cells)	10 µM

**Human fetal tissue derived lung organoids (FLO) and LTFO medium**	
**Component**	**Final Concentration**
Advanced DMEM (Thermo 12634)	To volume
HEPES (Thermo 15630080)	10 mM
Glutamax (Thermo 35050061)	2 mM (1X)
Pen/Strep (Thermo 15140122)	1%
B-27 supplement minus Vitamin A (Thermo 12587010)	1X
n-acetylcysteine (Sigma A9165)	1.25 mM
N2 (Thermo 17502048)	1X
R-spondin 1 (Peprotech 120-38)	100 ng/mL
Noggin (Peprotech 120-10C)	100 ng/mL
EGF (Thermo PMG8043)	50 ng/mL
FGF10 (Peprotech 100-26)	100 ng/mL
FGF7 (Peprotech 100-19)	100 ng/mL
TGFb inhibitor (SB 431542) (Tocris 1614)	10 µM
GSK-3 inhibitor (CHIR 99021) (Tocris 4423)	3 µM
ROCK inhibitor Y-27632 (Tocris 1254) (Add to single cells)	10 µM

**Human fetal tissue derived small intestinal organoids (SiAFO) medium**	
**Component**	**Final Concentration**
Advanced DMEM (Thermo 12634)	To volume
HEPES (Thermo 15630080)	10 mM
Glutamax (Thermo 35050061)	2 mM (1X)
Pen/Strep (Thermo 15140122)	1%
B-27 supplement minus Vitamin A (Thermo 12587010)	1X
n-acetylcysteine (Sigma A9165)	1.25 mM
Wnt-3A (Peprotech 315-20)	100 ng/mL
R-spondin 1 (Peprotech 120-38)	500 ng/mL
Noggin (Peprotech 120-10C)	100 ng/mL
EGF (Thermo PMG8043)	50 ng/mL
Gastrin (Sigma G9020)	10 nM
TGFb inhibitor (A83-01) (Sigma SML0788)	500 nM
GSK-3 inhibitor (CHIR 99021) (Tocris 4423)	3 µM
P38 inhibitor (SB202190) (Sigma S7067)	10 µM
Prostaglandin E2 (Cambridge cay14010)	10 nM
ROCK inhibitor Y-27632 (Tocris 1254) (Add to single cells)	10 µM

Supplementary Table 3: media composition

Human SiAFO maturation medium	
**Component**	**Final Concentration**
Advanced DMEM (Thermo 12634)	To volume
HEPES (Thermo 15630080)	10 mM
Glutamax (Thermo 35050061)	2 mM (1X)
Pen/Strep (Thermo 15140122)	1%
B-27 supplement (Thermo 17504044)	1X
Nicotinamide (Sigma N0636)	10 mM
n-acetylcysteine (Sigma A9165)	1 mM
Wnt-3A (Peprotech 315-20)	0.1 nM
R-spondin from 293T-HA-Rspol-Fc cells	20% vol/vol
Noggin from HEK293 mNoggin-producing cells	10% vol/vol
EGF (Thermo PMG8043)	50 ng/mL
Gastrin (Sigma G9020)	10 nM
TGFb inhibitor (A83-01) (Tocris 2939)	500 nM
GSK-3 inhibitor (CHIR 99021) (Tocris 4423)	3 µM
P38 inhibitor (SB202190) (Sigma S7067)	10 µM
ROCK inhibitor Y-27632 (Tocris 1254) (Add to single cells)	10 µM

Human fetal tissue derived kidney organoid (FKO) medium	
**Component**	**Final Concentration**
Advanced DMEM (Thermo 12634)	To volume
HEPES (Thermo 15630080)	10 mM
Glutamax (Thermo 35050061)	2 mM (1X)
Pen/Strep (Thermo 15140122)	1%
B-27 supplement minus Vitamin A (Thermo 12587010)	1X
n-acetylcysteine (Sigma A9165)	1 mM
R-spondin 1 (Peprotech 120-38)	100 ng/mL
EGF (Thermo PMG8043)	50 ng/mL
TGFb inhibitor (A83-01) (Sigma SML0788)	2 µM
GSK-3 inhibitor (CHIR 99021) (Tocris 4423)	3 µM
FGF10 (Peprotech 100-26)	100 ng/mL
GDNF (Peprotech 450-10)	50 ng/mL
Heparin (Sigma H3393)	1 µg/mL
LDN193189 dihydrochloride (Cambridge Bioscience SM23-5)	200 nM
ROCK inhibitor Y-27632 (Tocris 1254) (Add to single cells)	10 µM

Human fetal tissue derived stomach organoid medium	
**Component**	**Final Concentration**
Advanced DMEM (Thermo 12634)	To volume
HEPES (Thermo 15630080)	10 mM
Glutamax (Thermo 35050061)	2 mM (1X)
Pen/Strep (Thermo 15140122)	1%
B-27 supplement minus Vitamin A (Thermo 12587010)	1X
n-acetylcysteine (Sigma A9165)	1.25 mM
Wnt-3A (Peprotech 315-20)	100 ng/mL
R-spondin 1 (Peprotech 120-38)	500 ng/mL
Noggin (Peprotech 120-10C)	100 ng/mL
EGF (Thermo PMG8043)	50 ng/mL
Gastrin (Sigma G9020)	10 nM
FGF10 (Peprotech 100-26)	200 ng/mL
TGFb inhibitor (A83-01) (Sigma SML0788)	5 µM
GSK-3 inhibitor (CHIR 99021) (Tocris 4423)	3 µM
ROCK inhibitor Y-27632 (Tocris 1254) (Add to single cells)	10 µM

Supplementary Table 3: media composition

Human distal tubule/collecting duct kidney differentiation medium	
**Component**	**Final Concentration**
Advanced DMEM (Thermo 12634)	To volume
HEPES (Thermo 15630080)	10 mM
Glutamax (Thermo 35050061)	2 mM
Pen/Strep (Thermo 15140122)	1%
Aldosterone (Sigma A9477)	10 nM
Vasopressin (Sigma V9879)	10 nM

Human distal lung medium	
**A) cSFDM (Complete serum free differentiation media)**	
**Component**	**Final Concentration**
IMDM (Thermo 12440053)	75%
Ham's F12 (Cellgro 10-080-CV)	25%
B-27 (with RA) supplement (Invitrogen 1750444)	1%
N-2 supplement (Invitrogen 17502048)	0.50%
BSA (Sigma A7030)	0.05%
Primocin (Thermo NC9392943)	100 µg/mL
Glutamax (Thermo 35050061)	1X
Ascorbic Acid (Sigma A4544)	50 µg/mL
MTG (Sigma M6145)	450 µM
**B) CK + DCI media**	
**Component**	**Final Concentration**
cSFDM Base	To volume
GSK-3 inhibitor (CHIR 99021) (Tocris 4423)	3 µM
FGF7 (Peprotech 100-19)	10 ng/mL
Dexamethasone (Sigma D4902)	50 nM
8BrcAMP (Sigma B7880)	0.1 mM
IBMX (Sigma I5879)	0.1 mM

Supplementary Table 4: List of antibodies

Antibody/conjugated molecules	Dilution
EpCAM (Abcam ab71916)	1:100
E-cadherin (BD 610182)	1:200
PDGF Receptor α (Cell signaling 3174)	1:200
Integrin β-4 (Abcam ab110167)	1:100
Integrin β-1 (Abcam 24693)	1:100
Zonula occludens-1 (Invitrogen 40-2200)	1:100
Pan Cytokeratin (Abcam ab7753)	1:100
Ki-67 (Abcam ab15580)	1:100
Ki-67 (Invitrogen 14-5698-82)	1:200
Cleaved Caspase-3 (Cell Signaling 9661)	1:100
Olfactomedin 4 (Cell Signaling 14369T)	1:50
Cytokeratin 20 (Proteintech 60183-1-Ig)	1:100
Lysozyme (Biorad 5790-4110)	1:50
Fatty Acid Binding Protein 1 (R&D AF1565)	1:100
Chromogranin A (Abcam ab15160)	1:1000
Mucin 2 (Santa Cruz sc-15334)	1:200
PAX8 (Abcam ab191870)	1:200
LIM1/LHX1 (Abcam ab229474)	1:100
GATA-3 (R&D AF2605)	1:200
Lotus Tetragonolobus Lectin, Fluorescein (Vector Laboratories FL-1321-2)	1:300
Acetylated α Tubulin (Santacruz sc-23950)	1:200
Calbindin 1 (Abcam ab108404)	1:100
SLC12A1 (Abcam ab171747)	1:100
Aquaporin-2 (Biotechne NB110-74682)	1:300
RET (R&D AF1485)	1:100
TTF1 (NKX2-1) (Abcam ab76013)	1:200
P63 (Abcam ab53039)	1:100
SOX2 (Abcam ab97959)	1:200
SOX2 (R&D AF2018)	1:200
SOX9 (R&D AF3075)	1:100
SOX9 (Merck ab5535)	1:100
Prosurfactant Protein C (Merck AB3786)	1:500
FOXJ1 (R&D AF3619)	1:100
Keratin 5 (BioLegend 905501)	1:100
Mucin 5AC (Invitrogen MA5-12178)	1:100
Surfactant Protein B (Thermo PA5-42000)	1:500
Phalloidin 488 (Thermo A12379)	1:200
Phalloidin 647 (Sigma Aldrich 65906)	1:200
Alexa Fluor Donkey anti-Mouse 488 (Thermo A21202)	1:500
Alexa Fluor Goat anti-Rabbit 488 (Thermo A11008)	1:500
Alexa Fluor Donkey anti-Rabbit (Thermo A21206)	1:500
Alexa Fluor Donkey anti-Rabbit 568 (Thermo A10042)	1:500
Alexa Fluor Donkey anti-Mouse 546 (Thermo A10036)	1:500
Alexa Fluor Donkey anti-Goat 633 (Thermo A21082)	1:500
Alexa Fluor Donkey anti-Rabbit 647 (Thermo A31573)	1:500
Alexa Fluor Goat anti-Rabbit 647 (Thermo A21244)	1:500
DyLight Donkey anti-Rat 550 (Thermo SA5-10027)	1:500
Hoechst 33342 (Thermo H1399)	1:500

Supplementary Table 5: List of primers

Primer Name	Forward	Reverse
human β-ACTIN	ATGGTGGGCATGGGTCAGA	GCAACGTACATGGCTGGGG
human OLFM4	CTTTCCAAAGTGAGGGAATATGTC	GATGTCAATTCGGACAGTTAGG
human LGR5	CAGTGCAGTGTTCACCTTCC	AGTGCCAGAACTGCTATGGT
human LYZ	CATTGTTCTGGGGCTTGTCC	TCATTACACCAGTAGCGGCT
human CHGA	GAAGAAGGCCCCACTGTAGT	AGTGCTCCTGTTCTCCCTTC
human MUC2	CAACAACTCCGAAGCTGTG	CAAATGTTTCTCGGTCACC
human ALPI	TCATCATGAGGGTGTGGCTT	TGTAGGCTTTGCTGTCCTGA
human FABP1	AAGACAGTGGTTCAGTTGGAAG	TGAGTTCGGTCACAGACTTGAT
human HES1	AGCACACTTGGGTCTGTGC	TGAAGAAAGATAGCTCGCGG
human AXIN2	GACAGGAATCATTCGGCCAC	CCTTCAGCATCCTCCGGTAT
human DLL1	ACTCCTACCGCTTCGTGTGT	CAGGGTTGCACACTTTCTCC
human ATOH1	GCAGGAGGAAAACAGCAAAA	ACTTGCCTCATCCGAGTCAC
