# Supplementary material for: Single-cell guided prenatal derivation of primary fetal epithelial organoids from human amniotic and tracheal fluids
Source: Nat Med. 2024 Mar 4;30(3):875–87. doi: 10.1038/s41591-024-02807-z (PMC10957479; doi:10.1038/s41591-024-02807-z)
Supplement: Supplementary file 2 — Reporting Summary [file 41591_2024_2807_MOESM2_ESM.pdf]

# nature portfolio

Corresponding author(s): Paolo De Coppi, Mattia Francesco Maria Gerli

Last updated by author(s): Dec 18, 2023



# Reporting Summary

Nature Portfolio wishes to improve the reproducibility of the work that we publish. This form provides structure for consistency and transparency in reporting. For further information on Nature Portfolio policies, see our Editorial Policies and the Editorial Policy Checklist.

## Statistics

For all statistical analyses, confirm that the following items are present in the figure legend, table legend, main text, or Methods section.

| n/a | Confirmed | |
|---|---|---|
| ☐ | ☒ | The exact sample size (*n*) for each experimental group/condition, given as a discrete number and unit of measurement |
| ☐ | ☒ | A statement on whether measurements were taken from distinct samples or whether the same sample was measured repeatedly |
| ☐ | ☒ | The statistical test(s) used AND whether they are one- or two-sided<br>*Only common tests should be described solely by name; describe more complex techniques in the Methods section.* |
| ☒ | ☐ | A description of all covariates tested |
| ☐ | ☒ | A description of any assumptions or corrections, such as tests of normality and adjustment for multiple comparisons |
| ☐ | ☒ | A full description of the statistical parameters including central tendency (e.g. means) or other basic estimates (e.g. regression coefficient) AND variation (e.g. standard deviation) or associated estimates of uncertainty (e.g. confidence intervals) |
| ☐ | ☒ | For null hypothesis testing, the test statistic (e.g. *F*, *t*, *r*) with confidence intervals, effect sizes, degrees of freedom and *P* value noted<br>*Give P values as exact values whenever suitable.* |
| ☒ | ☐ | For Bayesian analysis, information on the choice of priors and Markov chain Monte Carlo settings |
| ☒ | ☐ | For hierarchical and complex designs, identification of the appropriate level for tests and full reporting of outcomes |
| ☒ | ☐ | Estimates of effect sizes (e.g. Cohen's *d*, Pearson's *r*), indicating how they were calculated |

*Our web collection on statistics for biologists contains articles on many of the points above.*

## Software and code

Policy information about availability of computer code

| | |
|---|---|
| Data collection | Image data: Zeiss ZEN (v. 3.1), Nikon NIS-Elements (v. 5.41.02), Radius (v. 2.0), Beamline I13-1 (coherence branch) of the Diamond Light Source (Didcot, UK).<br>FACS data: BD FACSDiva (v. 8.0.1).<br>Plate reader data: SoftMax Pro (v. 7.1.2).<br>Bulk RNA sequencing data: NEBNext Low Input RNA library preparation was carried out and single-end sequencing completed on an Illumina NextSeq 2000 with 100 cycles.<br>Single cell RNA sequencing data: Single-cell libraries were constructed using the Chromium Next GEM Single Cell 3′ Reagent Kits v3.1 (Dual Index) from 10X Genomics, and were sequenced single-end on an Illumina NovaSeq 6000. |
| Data analysis | The code used for this analysis has been made publicly available on Zenodo (https://doi.org/10.5281/zenodo.8124205).<br><br>Data and statistical analyses were performed using the following softwares:<br><br>For analysis of fetal fluid-derived organoids Bulk RNA Sequencing data:<br>v2.20 bcl2fastq was used to convert Illumina novaSeq base call (BCL) files into FASTQ files.<br>v0.6.6 TrimGalore! was used to trim low quality reads (quality 20, length 70).<br>v2.7.1a STAR was applied to align the FASTQ sequences to the NCBI human reference genome GRCh38.p13.<br>v1.6.3 featureCounts quantified the expression of individual genes to generate the raw count matrix, using the GRCh38.104 gene annotation.<br>V2.48.3 biomaRt was used to assign Gene IDs.<br>v3.36.0 edgeR was used to normalise the data to counts per million (CPM).<br>V4.1.2 stats was used to calculated principle component analysis (PCA) on log2(CPM+1). |

The ComBat_seq function from v3.40.0 SVA was used for batch correction.
v1.32.0 DESeq2 was used to calculate differentially expressed genes (DEGs) and filtering was based on an adjusted p-value of <0.05 or <0.01 (see figure legend) and a positive Log Fold Change of >1 or >2 (see figure legend).
v3.3.6 ggplot2 for generation of dotplots showing CPM values and volcano plots showing up- and down-regulated DEGs.
v3.5 Metascape was used for pathway identification based on DEGs and v3.3.6 ggplot2 was used for their visualisation.
v1.0.12 pheatmap was used to generate hierarchical clustering heatmaps of samples

For analysis of single-cell RNA Sequencing AF and organoid cells data:
v2.20 bcl2fastq was used to convert Illumina novaSeq base call (BCL) files into FASTQ files.
v6.0.1 CellRanger was used to process the FASTQ files into count matrices.
v0.2.2 CellBender was used to reduce ambient RNA effects (only for AF cells)
v1.2.0 cellsnp-lite used for sample SNPs deconvolution
v0.5.6 vireo used for sample SNPs deconvolution
v4.1.1 Seurat was used to combine matrices and carry out normalisation.
v4.1.1 Seurat was used for batch correction (IntegrateData function), scaling (ScaleData), calculation of PCA (RunPCA), UMAP (runUMAP), and clustering (FindClusters).
v1.6.1 SingleR was used to label the epithelial cluster, in conjunction with investigation of epithelial specific markers.
v3.3.6 ggplot2 was used in R (v4.1.2) for generation of violin plots and dotplots.

R (v. 4.1.2) was used for generating and analysing all the single cell and bulk RNA Sequencing data.
Graph Pad Prism (v. 10.0.0) and Microsoft Excel (v. 16.67) were used to plot graphs, charts and conduct statistical analysis.
Images were processed and analysed using Fiji/ImageJ (v. 2.1.0) and Imaris (v. 8.2).
X-Ray PC-CT images were processed and analysed using Savu (DOI:https://doi.org/10.1016/j.softx.2022.101157) and Drishit (v. 2.6.4).
Micro-CT images were processed using modified Feldkamp filtered back projection algorithms with CTPro3D (Nikon, Metrology v. XT 5.1.43) and post-processed using VGStudio MAX (Volume Graphics GmbH, v. 3.4).
High-speed camera cilia video analysis was performed using IDT Motion Studio (v. 2.16.0.5.00).
TEM images were collected and processed using Radius (v. 2.0, EMSIS).
FACS analysis was performed using FlowJo (v. 10.15).
Schematic cartoons were generated using full-licensed BioRender and Servier Medical Art.

Numerosity and statistical test used are provided in the main text and in each figure legend.

For manuscripts utilizing custom algorithms or software that are central to the research but not yet described in published literature, software must be made available to editors and reviewers. We strongly encourage code deposition in a community repository (e.g. GitHub). See the Nature Portfolio guidelines for submitting code & software for further information.

# Data

Policy information about availability of data

All manuscripts must include a data availability statement. This statement should provide the following information, where applicable:

- Accession codes, unique identifiers, or web links for publicly available datasets
- A description of any restrictions on data availability
- For clinical datasets or third party data, please ensure that the statement adheres to our policy

Raw individual-level data and combined processed data of the bulk RNA sequencing (AFO, TFO, Fetal tissue-derived organoids) and scRNAseq (AF, AFO) have been uploaded to the NCBI GEO public repository (GSE220994). This data is openly available with no restriction or time limit. Questions or additional requests can be directed to the corresponding authors.

Additional datasets or references used in this study can be found at:
- NCBI human reference genome GRCh38.p13 - ncbi.nlm.nih.gov/datasets/genome/GCF_000001405.39/
- The National Center for Biotechnology Information (NCBI) Human SNPs dataset - ftp://ftp.ncbi.nlm.nih.gov/snp/organisms/human_9606/VCF/00-common_all.vcf.gz
- Primary Human Cell Atlas Data - Mabbott, N.A., Baillie, J.K., Brown, H. et al. An expression atlas of human primary cells: inference of gene function from coexpression networks. BMC Genomics 14, 632 (2013). https://doi.org/10.1186/1471-2164-14-632
- C8 cell type signature gene sets used for scGSEA annotation of scRNAseq - gsea-msigdb.org/gsea/msigdb/
- Cells_Intestinal_Tract - doi.org/10.1038/s41586-021-03852-1 - celltypist.org/models
- Cells_Fetal_Lung - doi.org/10.1016/j.cell.2022.11.005 - celltypist.org/models
- Cells_Lung_Airway - doi.org/10.1101/2021.11.26.470108 - celltypist.org/models
- Human_Lung_Atlas - doi.org/10.1101/2022.03.10.483747 - celltypist.org/models
- The integrated Human Lung Cell Atlas (HLCA) v1.0 - doi.org/10.1038/s41591-023-02327-2 - data.humancellatlas.org/hca-bio-networks/lung
- DevKidCC - doi.org/10.1186/s13073-022-01023-z; doi.org/10.1371/journal.pbio.3000152; doi.org/10.1681/ASN.2017080890

# Human research participants

Policy information about studies involving human research participants and Sex and Gender in Research.

| | |
|---|---|
| Reporting on sex and gender | The sex of the specimens was not considered as a parameter for the study design. Both male and female samples were included in the single cell RNA sequencing or used for organoids derivation and analysis. A detailed breakdown is presented in Supplementary Table 1. |
| Population characteristics | Patients characteristic are highlighted in Supplementary Table 1, whenever these were available to our knowledge. |

Human fetal fluid (AF/TF) samples were collected from pregnancies ranging 15-34 gestational age weeks (GA).

Human fetal tissue derived cells were isolated from tissue samples procured through the MRC/Wellcome Trust Human Developmental Biology Resource (HDBR) and ranged from 11 to 23 GA.

Pediatric intestinal ileal sample was collected from a 3 years-old patient (M) with Hirschprung's disease.

Recruitment

Fetal post-mortem tissue samples were sourced following informed consent via the Joint MRC/Wellcome Trust Human Developmental Biology Resource (HDBR) with Research Tissue Bank ethical approval.

Relevant patients listed for amniocentisis, amniodrainage or surgery procedures were identified by the UCLH and UZ Leuven clinical teams, which were separate from the laboratory research team. The parents or guardians of the patients were approached by a member of the clinical team. Once informed consent was gained, the fluid was collected and transfered to the research team for processing for experiments.

The patients and the parents or guardians of the patients for collection of pediatric intestinal tissue were approached by a member of the clinical team. Once informed consent was gained, the tissue was collected and transfered to the research team for processing for experiments.

Ethics oversight

Ethical oversight was provided by the NHS Health Research Authority, in accordance with the Governance Arrangements for Research Ethics Committees and complied fully with the Standard Operating Procedures for Research Ethics Committees in the UK and Belgium

Fetal fluid samples were collected from the University College London Hospital (UCLH) Fetal Maternal Unit (FMU) (REC 14/LO/0863 IRAS 133888) and UZ Leuven (Ethics committee number S53548) as part of standard patient's clinical care.

Fetal tissue samples were sourced via the Joint MRC/Wellcome Trust Human Developmental Biology Resource under informed ethical consent with Research Tissue Bank ethical approval (Project 200478: UCL REC 18/LO/0822 - IRAS ID 244325; Newcastle 18/NE/0290 - IRAS ID 250012).

Pediatric intestinal tissue sample was obtained upon informed consent and ethical approval for the use of human tissue obtained from the East of England - Cambridge Central Research Ethics Committee (REC reference 18/EE/0150). The Committee was constituted in accordance with the Governance Arrangements for Research Ethics Committees and complied fully with the Standard Operating Procedures for Research Ethics Committees in the UK.

Note that full information on the approval of the study protocol must also be provided in the manuscript.

# Field-specific reporting

Please select the one below that is the best fit for your research. If you are not sure, read the appropriate sections before making your selection.

☒ Life sciences ☐ Behavioural & social sciences ☐ Ecological, evolutionary & environmental sciences

For a reference copy of the document with all sections, see nature.com/documents/nr-reporting-summary-flat.pdf

# Life sciences study design

All studies must disclose on these points even when the disclosure is negative.

Sample size

Sample size for every experiment performed and image acquired is presented in the figure legends. Supplementary table 1 also provides a detailed list of all the amniotic and tracheal fluid samples used in the study and the experiments performed on each sample. Further information on sample size (e.g. number of cells and organoids used for analysis) are detailed in Supplementary Table 2.

For imaging data, images in the manuscript are representative of a minimum n=3 experiments. In some cases sample size was determined by the availability of patient samples. For instance: Figure 3 n=2 biological samples; All the sample sizes are stated in each figure legend.

For quantitative analyses, no sample size calculation was performed but the sample size / replicate number was chosen in order to provide sufficient data points for the determination of measures of central tendency, variance, and parametric vs non-parametric distribution of the data whenever possible.

Statistical significance of reported results was assessed by statistical tests during data analyses, as indicated in Methods section. Statistical significance is stated in each figure legend.

Data exclusions

2 organoid samples were removed from the analysis due to poor sequencing quality. Along with our analysis we included controls deemed to help with QC and batch correction. With this aim we have sequenced 1 cell line of human mesenchymal cells (mesoangioblasts, MABSCT); 1 organoid line was repeatedly sequenced across different batches. These samples were removed from the final analysis and were not uploaded in the dataset on NCBI GEO.

1 organoid line resulted in a mixed renal and pulmonary phenotype at the transcriptomic level. We ascribed this to a possible technical error of the operator during organoid picking. This sample was labeled as unknown and only presented in Extended Data Fig.2f.

No further data were excluded from the analyses.

| | |
|---|---|
| Replication | Cell culture experiments were replicated within our laboratory by at least 5 independent operators (G.C., B.S., G.G.G, B.C.J and K.Y.S) with a minimum of n=3 technical replicates per experiment. Moreover, the intestinal expansion and maturation experiments presented in Figure 3 were conducted in parallel and independently in 3 different laboratories (P.D.C., J.D. and V. SW. L)<br><br>All attempts at replication were successful. Derivation of organoids was not successful for all the samples as stated in the main text and in Extended Data Fig.2c. We ascribed this on inter biological variability across individuals. |
| Randomization | Randomization was only applied for assembling the dot plots presented in Figures 4b and 5b. Organoids showed were randomly selected from the entire pool of sequenced organoids. However, a full list of the organoids sequenced was plotted and presented in Extended Data Figures 4b and 5d.<br><br>For all other analyses, organoids were only grouped based on previously known characteristics or based on the organoid identity, as determined during the research. Any and all grouping is made clear throughout the manuscript and within the metadata available through the GEO accession |
| Blinding | The investigators analyzing the experiment were not the same investigators performing the experiment for all immunofluorescence, RNA Seq data, functional, and qPCR data. Quantifications were performed in blind from different operators. During data collection, samples were given codes to blind the analyzing investigator from group allocation. RNAseq data analysis was done blinded independently from the investigator who performed experiment to conceal their group allocations. Blinding was only performed in the first stage of the data quantification/ analysis, then it was removed to allow data discussion and contribution of all the investigators involved. |

# Reporting for specific materials, systems and methods

We require information from authors about some types of materials, experimental systems and methods used in many studies. Here, indicate whether each material, system or method listed is relevant to your study. If you are not sure if a list item applies to your research, read the appropriate section before selecting a response.

## Materials & experimental systems

| n/a | Involved in the study | |
|---|---|---|
| ☐ | ☒ | Antibodies |
| ☐ | ☒ | Eukaryotic cell lines |
| ☒ | ☐ | Palaeontology and archaeology |
| ☒ | ☐ | Animals and other organisms |
| ☒ | ☐ | Clinical data |
| ☒ | ☐ | Dual use research of concern |

## Methods

| n/a | Involved in the study | |
|---|---|---|
| ☒ | ☐ | ChIP-seq |
| ☐ | ☒ | Flow cytometry |
| ☒ | ☐ | MRI-based neuroimaging |

## Antibodies

Antibodies used

Antibody/conjugated molecules and Dilution

• EpCAM (Abcam ab71916) 1:100
- abcam.com/products/primary-antibodies/epcam-antibody-ab71916.html#description_references
• E-cadherin (BD 610182) 1:200
- bdbiosciences.com/en-eu/products/reagents/microscopy-imaging-reagents/immunofluorescence-reagents/purified-mouse-anti-e-cadherin.610182#citations_references
• PDGF Receptor α (Cell signaling 3174) 1:200
- cellsignal.com/products/primary-antibodies/pdgf-receptor-a-d1e1e-xp-rabbit-mab/3174#pdpCiteABCitations
• Integrin β-4 (Abcam ab110167) 1:100
- abcam.com/products/primary-antibodies/integrin-beta-4-antibody-439-9b-ab110167.html#description_references
• Integrin β-1 (Abcam 24693) 1:100
- abcam.com/products/primary-antibodies/integrin-beta-1-antibody-p5d2-ab24693.html#description_references
• Zonula occludens-1 (Invitrogen 40-2200) 1:100
- thermofisher.com/antibody/product/ZO-1-Antibody-Polyclonal/40-2200#references-component-id
• Pan Cytokeratin (Abcam ab7753) 1:100
- abcam.com/products/primary-antibodies/pan-cytokeratin-antibody-c-11-ab7753.html#description_references
• Ki-67 (Abcam ab15580) 1:100
- abcam.com/products/primary-antibodies/ki67-antibody-ab15580.html#description_references
• Ki-67 (Invitrogen 14-5698-82) 1:200
- thermofisher.com/antibody/product/Ki-67-Antibody-clone-SolA15-Monoclonal/14-5698-82#references-component-id
• Cleaved Caspase-3 (Cell Signaling 9661) 1:100
- cellsignal.com/products/primary-antibodies/cleaved-caspase-3-asp175-antibody/9661#pdpCiteABCitations
• Olfactomedin 4 (Cell Signaling 14369T) 1:50
- cellsignal.com/products/primary-antibodies/olfm4-d1e4m-xp-rabbit-mab/14369#pdpCiteABCitations
• Cytokeratin 20 (Proteintech 60183-1-lg) 1:100
- ptglab.com/products/KRT20-Antibody-60183-1-Ig.htm#publications
• Lysozyme (Biorad 5790-4110) 1:50 - bio-rad-antibodies.com/monoclonal/human-lysozyme-antibody-sb1-bgn-06-961-5790-4110.html?f=purified#Datasheets
• Fatty Acid Binding Protein 1 (R&D AF1565) 1:100

- rndsystems.com/products/human-mouse-rat-fabp1-l-fabp-antibody_af1565#product-citations
• Chromogranin A (Abcam ab15160) 1:1000
- abcam.com/products/primary-antibodies/chromogranin-a-antibody-ab15160.html#description_references
• Mucin 2 (Santa Cruz sc-15334) 1:200
- scbt.com/p/mucin-2-antibody-h-300#citations
• PAX8 (Abcam ab191870)1:200
- abcam.com/en-sk/products/primary-antibodies/pax8-antibody-epr18715-ab191870#view=publications
• LIM1/LHX1 (Abcam ab229474) 1:100 - abcam.com/products/primary-antibodies/lim1lhx1-antibody-ab229474.html
• GATA-3 (R&D AF2605) 1:200
- rndsystems.com/products/human-gata-3-antibody_af2605#product-citations
• Acetylated α Tubulin (Santacruz sc-23950) 1:200
- scbt.com/p/acetylated-alpha-tubulin-antibody-6-11b-1#citations
• Calbindin 1 (Abcam ab108404) 1:100
- abcam.com/products/primary-antibodies/calbindin-antibody-ep3478-ab108404.html#description_references
• SLC12A1 (Abcam ab171747) 1:100
- abcam.com/products/primary-antibodies/slc12a1nkcc2-antibody-epr11842-ab171747.html#description_references
• Aquaporin-2 (Biotechne NB110-74682) 1:300
- novusbio.com/products/aquaporin-2-antibody_nb110-74682#PublicationSection
• RET (R&D AF1485) 1:100
- rndsystems.com/products/human-ret-antibody_af1485#product-citations
• TTF1 (NKX2-1) (Abcam ab76013) 1:200
- abcam.com/products/primary-antibodies/ttf1-antibody-ep1584y-ab76013.html#description_references
• P63 (Abcam ab53039) 1:100
- abcam.com/products/primary-antibodies/p63-antibody-ab53039.html#description_references
• SOX2 (Abcam ab97959) 1:200
- abcam.com/products/primary-antibodies/sox2-antibody-ab97959.html#description_references
• SOX2 (R&D AF2018) 1:200
- rndsystems.com/products/human-mouse-rat-sox2-antibody_af2018#product-citations
• SOX9 (R&D AF3075) 1:100
- rndsystems.com/products/human-sox9-antibody_af3075#product-citations
• SOX9 (Merck ab5535) 1:100
- merckmillipore.com/GB/en/product/Anti-Sox9-Antibody,MM_NF-AB5535#anchor_REF
• Prosurfactant Protein C (Merck AB3786) 1:500
- merckmillipore.com/GB/en/product/Anti-Prosurfactant-Protein-C-proSP-C-Antibody,MM_NF-AB3786#anchor_REF
• FOXJ1 (R&D AF3619) 1:100
- rndsystems.com/products/human-foxj1-antibody_af3619#product-citations
• Keratin 5 (BioLegend 905501) 1:100
- biolegend.com/Files/Images/media_assets/pro_detail/datasheets/905501-keratin-5-polyclonal-antibody-purified-10956-IFU-Rev-7.pdf?v=20220914082417
• Mucin 5AC (Invitrogen MA5-12178) 1:100
- thermofisher.com/antibody/product/MUC5AC-Antibody-clone-45M1-Monoclonal/MA5-12178#references-component-id
• Surfactant Protein B (Thermo PA5-42000) 1:500
- thermofisher.com/antibody/product/SFTPB-Antibody-Polyclonal/PA5-42000#references-component-id
• APC/Fire™ 750 anti-human CD324 (E-Cadherin) (Biolegend 324122) 5ul/tube
- biolegend.com/ja-jp/products/apcfire-750-anti-mouse-human-cd324-e-cadherin-16446?GroupID=GROUP20#productCitations
• APC/Fire™ 750 anti-human CD326 (EpCAM) (Biolegend 324233) 5ul/tube
- biolegend.com/en-gb/cell-separation/apc-fire-750-anti-human-cd326-epcam-antibody-13581?GroupID=BLG5134#productCitations

• Lotus Tetragonolobus Lectin, Fluorescein (Vector Laboratories FL-1321-2) 1:300
• Phalloidin 488 (Thermo A12379) 1:200
• Phalloidin 647 (Sigma Aldrich 65906) 1:200
• Alexa Fluor Donkey anti-Mouse 488 (Thermo A21202) 1:500
• Alexa Fluor Goat anti-Rabbit 488 (Thermo A11008) 1:500
• Alexa Fluor Donkey anti-Rabbit (Thermo A21206) 1:500
• Alexa Fluor Donkey anti-Rabbit 568 (Thermo A10042) 1:500
• Alexa Fluor Donkey anti-Mouse 546 (Thermo A10036) 1:500
• Alexa Fluor Donkey anti-Goat 633 (Thermo A21082) 1:500
• Alexa Fluor Donkey anti-Rabbit 647 (Thermo A31573) 1:500
• Alexa Fluor Goat anti-Rabbit 647 (Thermo A21244) 1:500
• DyLight Donkey anti-Rat 550 (Thermo SA5-10027) 1:500
• Hoechst 33342 (Thermo H1399) 1:500

A list of antibodies used in the study is also available in Supplementary Table 4

Validation

Antibodies were validated using validation information on the manufacturer's website. The antibodies listed in the methods section are widely used commercially available antibodies, and are validated by the companies with publications cited on the company websites. Above, we provide links to literature which also successfully used these antibodies.

Furthermore, in our experiments several antibodies were further validated 'in house' using human primary fetal tissue sections or primary fetal tissue derived organoids. Staining protocols included positive and negative controls where appropriate.

# Eukaryotic cell lines

Policy information about cell lines and Sex and Gender in Research

| | |
|---|---|
| Cell line source(s) | Human fetal cells were isolated from amniotic fluid and tracheal fluid obtained through amniocentesis, amniodrainage or fetal surgical procedure from pregnancies spanning from 15 to 34 gestational age weeks (GA). Human fetal tissue derived cells were isolated from 11 to 23 GA fetal specimens.<br><br>No other cell lines were used in this study. |
| Authentication | Cell and organoid lines were not authenticated. |
| Mycoplasma contamination | All cells and organoids used in this work were tested monthly and always tested negative for Mycoplasma |
| Commonly misidentified lines<br>(See ICLAC register) | No misidentified lines were used |

# Flow Cytometry

## Plots

Confirm that:

☒ The axis labels state the marker and fluorochrome used (e.g. CD4-FITC).

☒ The axis scales are clearly visible. Include numbers along axes only for bottom left plot of group (a 'group' is an analysis of identical markers).

☒ All plots are contour plots with outliers or pseudocolor plots.

☒ A numerical value for number of cells or percentage (with statistics) is provided.

## Methodology

| | |
|---|---|
| Sample preparation | For amniotic fluid viable cells isolation:<br>After collection, fluids were stored at 4°C until processing.  AF samples were passed through a 70 µm and 40 µm cell strainer and transferred in 50ml tubes before being centrifuged at 300 g for 10 min at 4°C. Supernatant was discarded, pellet resuspended in 5-10 mL of FACS blocking buffer containing 1% FBS and 0.5 mM EDTA in PBS and transferred to FACS tubes. Cells were incubated with 5 µg/mL Hoechst (Sigma-Aldrich, 33342) for 40 min at 37°C and then counterstained with 2 µg/mL propidium iodide (PI) (Sigma-Aldrich, P4170) for 5 min at RT. Viable cells were sorted using a FACSAria III (BD), unselected for side and forward scatter, but gated for Hoechst+ and PI-.<br>For follow on analysis the viable cells were:<br>Cells were incubated for 30 min at 4 degree with the following fluorochrome-conjugated antibodies: APC/Fire™ 750 anti-human CD324 (E-Cadherin) (Biolegend 324122), APC/Fire™ 750 anti-human CD326 (EpCAM) (324233). |
| Instrument | BD FACSAria III and BD FACSymphony A5 |
| Software | BD FACSDiva (v. 8.0.1), FlowJo (v. 10.15) |
| Cell population abundance | Viable cells represented 1-3% of the sorting events reported. Viability was confirmed using a fluorescent cell counter (Luna) using the Live/Dead kit (Acridine orange/Propidium iodide) |
| Gating strategy | To account for the large variability in size and shape of the cells present in the AF, prior sorting the cells were not subjected to a side and forward scatter gating (SSC/FSC). The population was gated for Hoechst (33342) positivity and for Propidium iodide negativity |

☒ Tick this box to confirm that a figure exemplifying the gating strategy is provided in the Supplementary Information.
