# Supplementary material for: Single-cell guided prenatal derivation of primary fetal epithelial organoids from human amniotic and tracheal fluids
Source: Nat Med. 2024 Mar 4;30(3):875–87. doi: 10.1038/s41591-024-02807-z (PMC10957479; doi:10.1038/s41591-024-02807-z)
Supplement: Supplementary file 7 — Flow cytometry data analysis. [file 41591_2024_2807_MOESM7_ESM.pdf]

ECAD

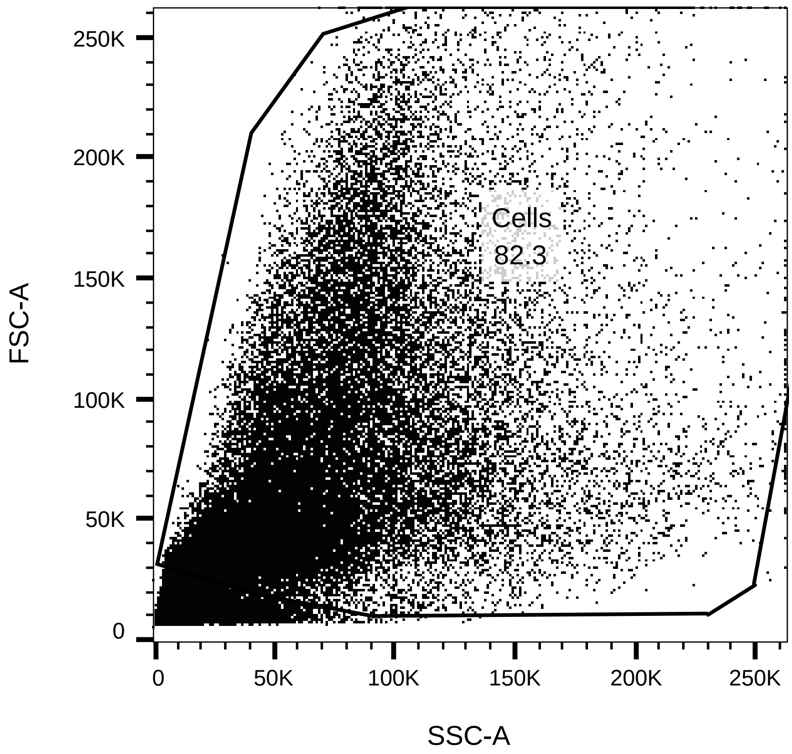

AF Epithelial Nature 14Aug_stained ECAD.fcs
Ungated
46633

	Sample Name	Subset Name	Count
(cyan)	AF Epithelial Nature 14Aug_unstained_001.fcs	Cells	34045
(red)	AF Epithelial Nature 14Aug_stained ECAD.fcs	Cells	38389

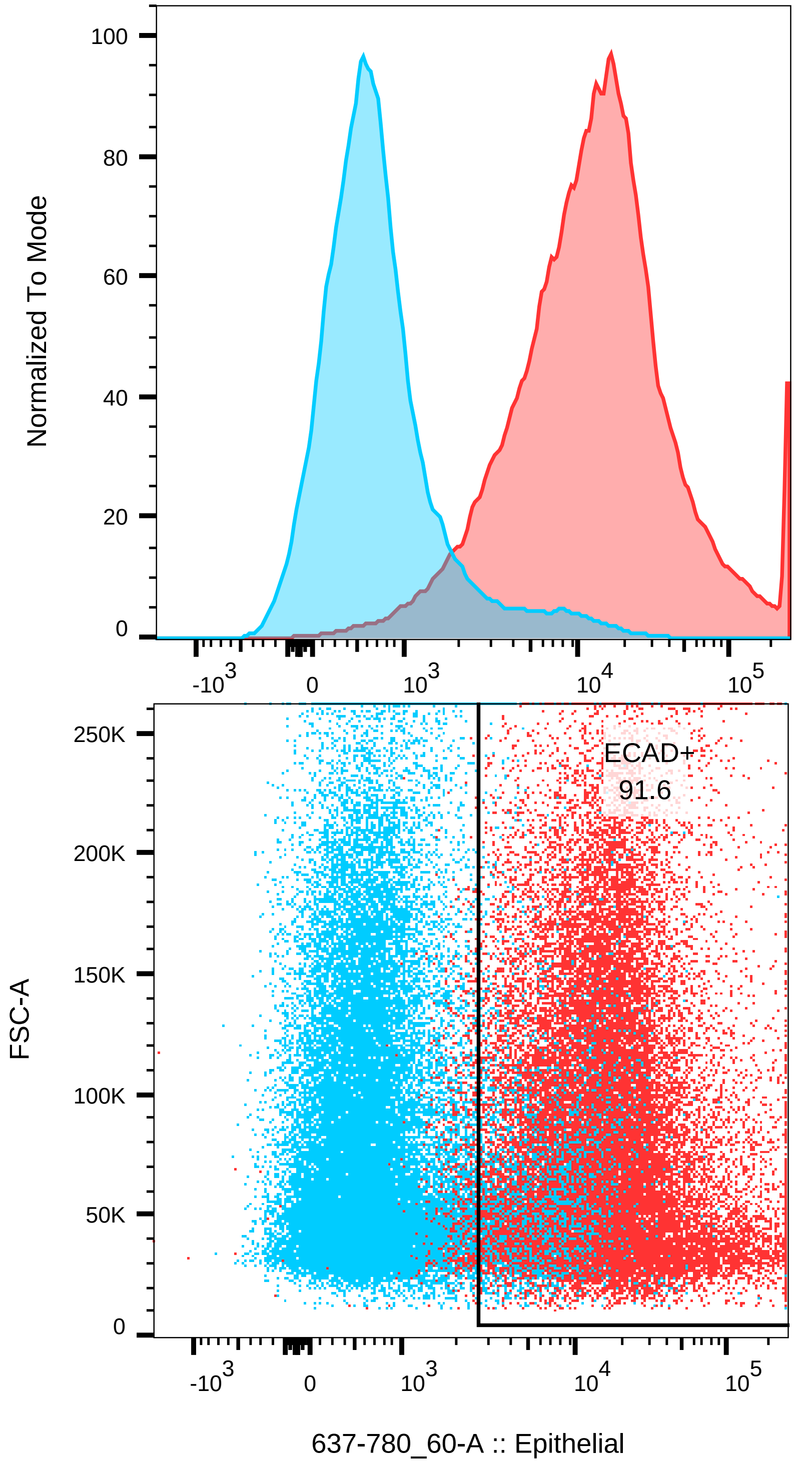

EpCAM

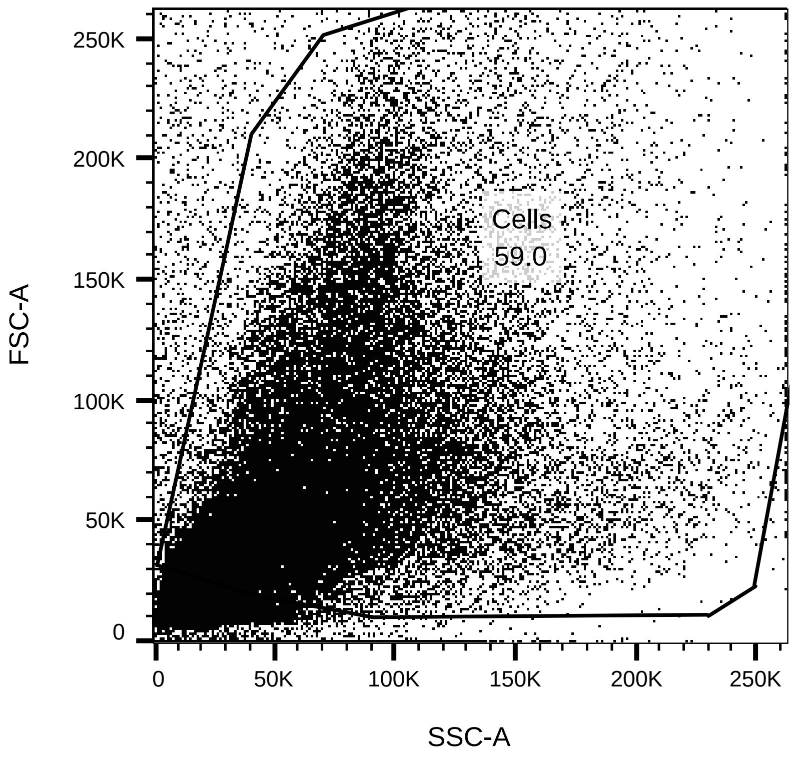

AF Epithelial Nature 14Aug_stained EPCAM.fcs
Ungated
100000

	Sample Name	Subset Name	Count
(cyan)	AF Epithelial Nature 14Aug_unstained_001.fcs	Cells	34045
(red)	AF Epithelial Nature 14Aug_stained EPCAM.fcs	Cells	58964

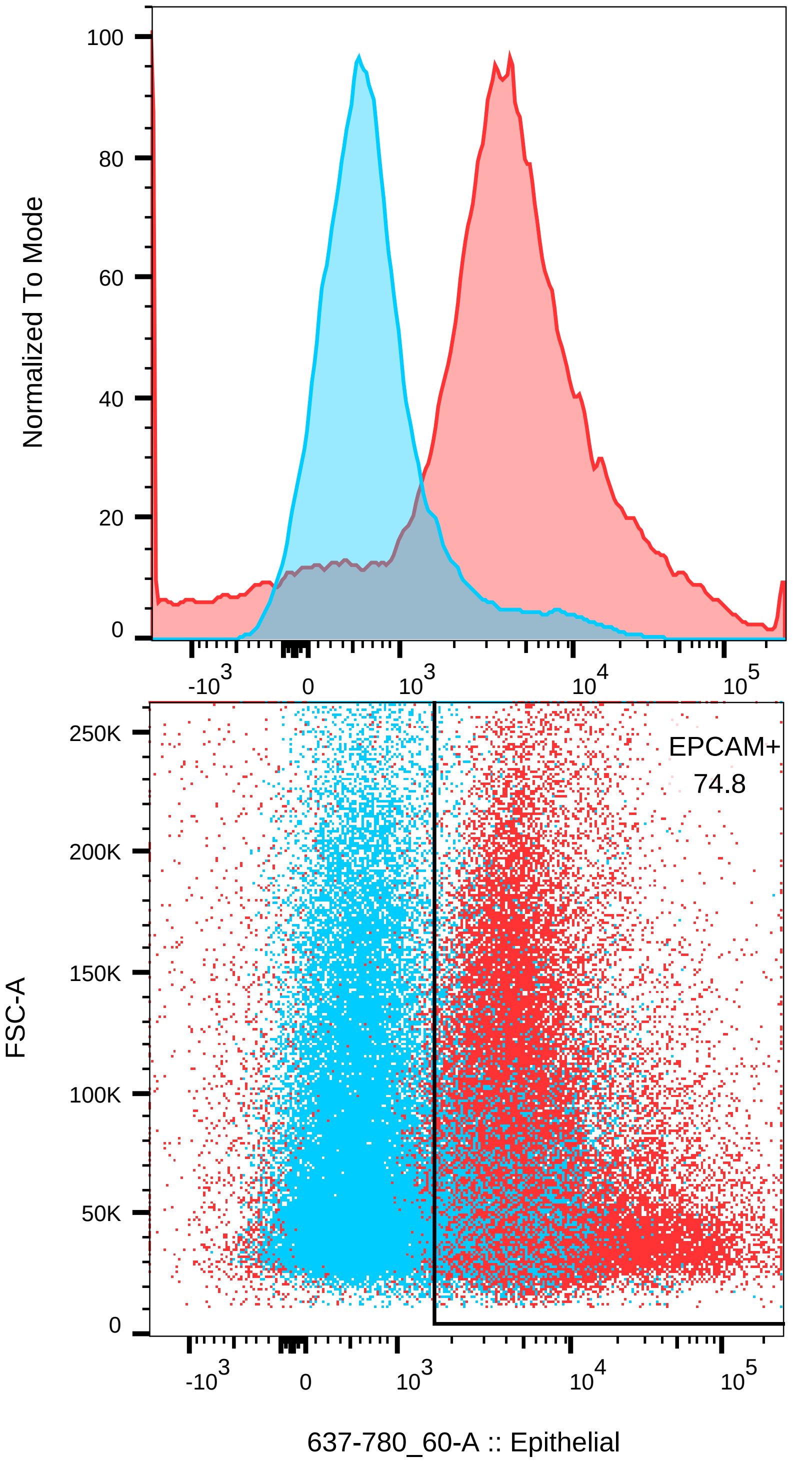

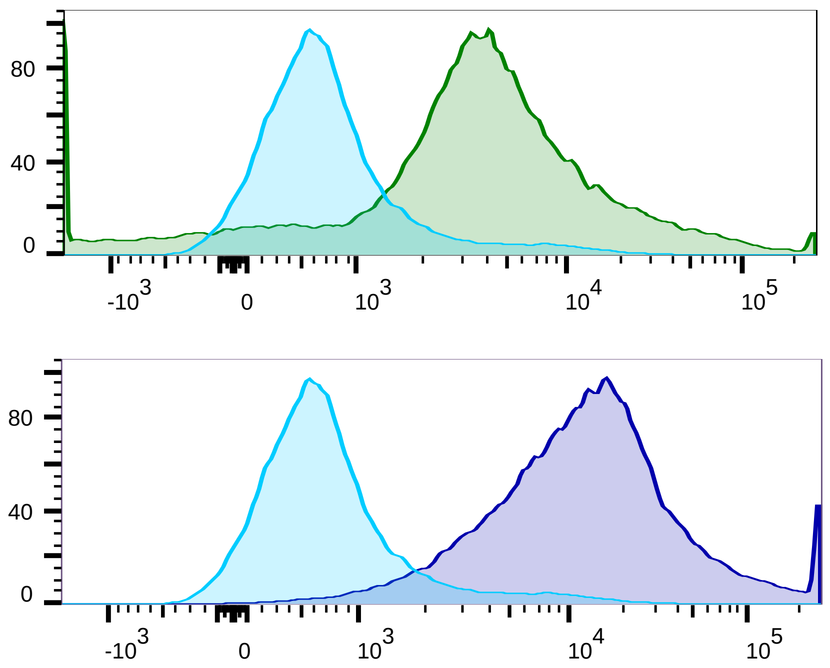

80
40
0
-10^3
0
10^3
10^4
10^5
80
40
0
-10^3
0
10^3
10^4
10^5
