# Supplementary figures and images for: Single-cell guided prenatal derivation of primary fetal epithelial organoids from human amniotic and tracheal fluids
Source: Nat Med. 2024 Mar 4;30(3):875–87. doi: 10.1038/s41591-024-02807-z (PMC10957479; doi:10.1038/s41591-024-02807-z)

UNSTAINED CT

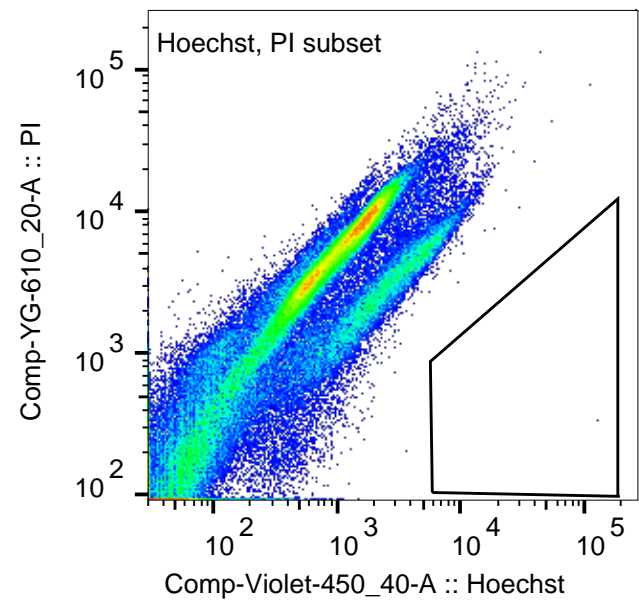

STAINED - VIABLE CELLS

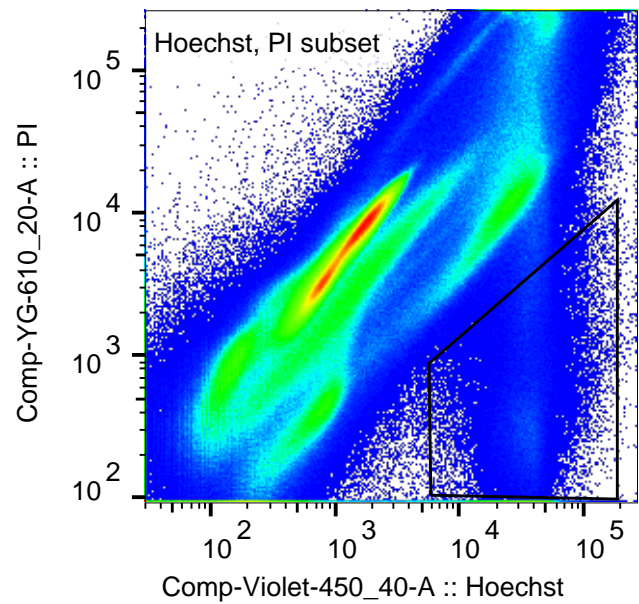

STAINED - VIABLE CELLS

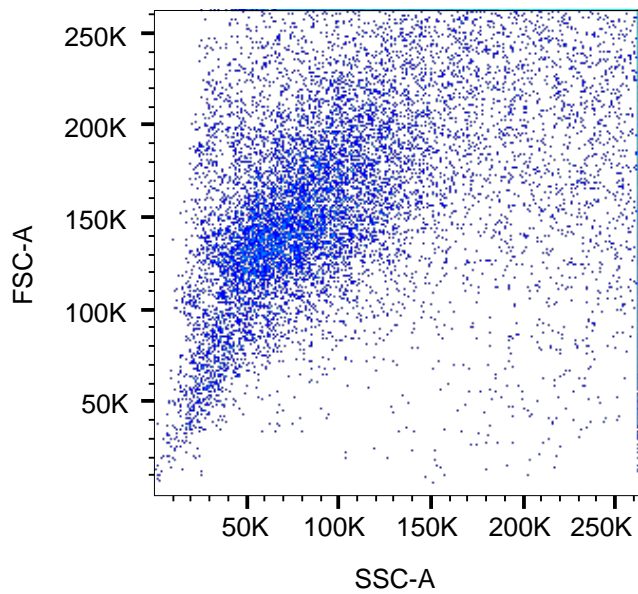

Supplement: Supplementary file 13 — FACS data analysis. [file 41591_2024_2807_MOESM13_ESM.pdf]
